# Synaptic Spine Head Morphodynamics from Graph Grammar Rules for Actin Dynamics

**Published:** 2025-04-18

**Authors:** Matthew Hur, Thomas Bartol, Padmini Rangamani, Terrence Sejnowski, Eric Mjolsness

**Affiliations:** Program in Mathematical, Computational, and Systems Biology, Univeristy of California Irvine, Irvine, CA, USA; Computational Neurobiology Laboratory, Salk Institute for Biological Studies, La Jolla, CA, USA; Departments of Pharmacology and Mechanical and Aerospace Engineering, University of California San Diego, La Jolla, CA, USA; Computational Neurobiology Laboratory, Salk Institute for Biological Studies, La Jolla, CA, USA; Deparment of Neurobiology, University of California San Diego, La Jolla, CA, USA; Departments of Computer Science and Mathematics, Univeristy of California Irvine, Irvine, CA, USA

## Abstract

There is a morphodynamic component to synaptic learning by which changes in dendritic (postsynaptic) spine head size are associated with the strengthening or weakening of the synaptic connection between two neurons, in response to the temporal correlation of local presynaptic and postsynaptic signals. These morphological factors are in turn sculpted by the dynamics of the actin cytoskeleton. In this paper, we use Dynamical Graph Grammars (DGGs) implemented within a computer algebra system to model how networks of actin filaments can dynamically grow or shrink, reshaping the spine head.

Dynamical Graph Grammars (DGGs) provide a well-defined way to accommodate dynamically changing system structure such as active cytoskeleton represented using dynamic graphs, within nonequilibrium statistical physics under the master equation. We show that DGGs can also incorporate biophysical forces between graph-connected objects at a finer time scale, with specialized DGG kinetic rules obeying biophysical constraints of Galilean invariance, conservation of momentum, and dissipation of conserved global energy. We use graph-local energy functions for cytoskeleton networks interacting with membranes, and derive DGG rules from the specialization of dissipative stochastic dynamics - separated into dissipative and thermal noise rule types - to a mutually exclusive and exhaustive collection of graph-local neighborhood types for the rule left hand sides. The dissipative rules comprise a stochastic version of gradient descent dynamics. The thermal noise rules use a Gaussian approximation of each position coordinate to sample jitter-like displacements.

For the spine head model we designed and implemented DGG grammar mathematical sub-models including actin network growth, non-equilibrium statistical mechanics, and filament-membrane mechanical interaction to regulate the re-writing of graph objects. We simulate emergent biophysics of simplified networks of actin polymers and their interactions with membranes. From a biological perspective, we observe regulatory effects of three actin-binding proteins (ABPs) on the membrane size and find evidence supporting mechanisms of membrane growth.

## 1 Introduction

Actin cytoskeletal dynamics leads to changes in the shape of cells through its action on the membranes of signal-receiving neuronal compartments called dendritic spine heads (Hotulainen and Hoogenraad [2010]). Synaptic spine heads influence learning because their size influences the strength of the synaptic connection between two neurons (Araya et al. [2014]). Repeated firing across the same synapse can lead to a long-lasting connection in the form of memory, as in Hebbian learning (Hebb [2002]). Models of the synaptic spine head hold potential to model behaviors such as addiction and have been posited to be explanatory for the engram hypothesis in how memory is stored in the brain (Bonilla-Quintana and Rangamani [2022], Lisman [2017], Lee et al. [2024]). Actin’s importance in synaptic biophysical kinetics can lead to therapeutics that target memory and learning (A et al. [2025]).

Actin filament biophysics can be modeled from principles that incorporate mechanical memory of signaling from neuron to neuron. The CaMKIIβ molecule is a bundling protein that strengthens individual chains of actins by cross-linking them (Okamoto et al. [2007]). Its binding to the filament form of actin (F-actin) thus leads to stronger filaments (Yasuda et al. [2022]). CaMKIIβ’s binding along actin filaments allows the intermolecular potentials to exert a force on the connected nodes. There have been several investigations into hysteresis in this system which is indicative of memory manifesting in cytoskeletal networks (Yasuda et al. [2022]). One experimental study illustrates how individual actin filaments bundle and incorporate memory in the form of a hysteretic response to repeated compression-extension cycles (Rückerl et al. [2017]). Another study considers how mechanical hysteresis arises in actin networks as a whole by their mechanism of memorizing the crosslinking-induced anisotropy both *in vitro* and *in silico* (Scheff et al. [2022]).

Prior actin models have been implemented in niche simulation packages such as Cytosim (Akamatsu et al. [2020]), but they make assumptions about biophysical filament systems that can be fully accounted for using a declarative and highly expressive simulation package based on DGGs (Mjolsness [2019]). Moving boundaries have been approached more frequently as part of continuum or hybrid models, e.g. partial differential equations (Bonilla-Quintana and Rangamani [2024]), finite element methods (Hernandez-Aristizabal et al. [2024]), or phase-field methods (Moure and Gomez [2016]). Yet, these approaches may lack the ability to impose complex biophysical constraints through simple local rules, or they lack fine-granularity in either the internal cytoskeleton or the forces onto the membrane mesh. What is needed is a graph-based approach that can downscale the granularity of the internal cytoskeleton representation and that enables easy expression of biophysical complexities such as membrane mesh dynamics including for example the Newtonian reaction forces between the cytoskeleton and membrane.

In order to simulate actin taking into account connectivity of an actin network, we build a model as a Dynamical Graph Grammar (DGG) (Mjolsness [2019]) implemented in a computer algebra system in which expressive rules are written for pattern matching in the underlying package. DGGs are theoretically grounded (Mjolsness [2022]) and originate as a graph-based notational extension of Stochastic Parameterized Grammars (SPGs) which, highly expressive by nature, is applied to node-labeled multisets instead of graphs, and incorporates probability distributions over rule firings and their outcomes (Mjolsness and Yosiphon [2006]). DGGs also incorporate differential equation dynamics. Here dozens of mathematical rules expressed in Plenum, a Mathematica package that implements DGGs, (Yosiphon [2009]), model the behavior of the actin network remodeling, biophysical kinetics, and interaction with membrane, while the membrane has its own rules for curvature, pressure, and tension updates. We show that we are able to simulate a dynamically changing cellular protrusion within reasonable time scales using the highly expressive DGG package Plenum in the Mathematica computer algebra system (Yosiphon [2009]).

Our work complements prior modeling of synaptic spine heads based on differential equations that model the actin cytoskeleton. Previously, there has been work on modeling actin cytoskeleton forces exerted onto synaptic spine membranes using differential equations (Bonilla-Quintana et al. [2020, 2021], Bonilla-Quintana and Rangamani [2024]). In our work, we implement the Brownian ratchet (BR) hypothesis which posits that fluctuations in both the lipid bilayer and ends of actin filaments lead to large enough distances between them for actin to polymerize (Mogilner and Oster [2003]). This polymerization then provides force onto the membrane. Previous models with a membrane coarse-grain membrane-filament interaction as a spring attachment without BR (Ni and Papoian [2021]) and others, along with the coarse inter-module interaction, use a partial membrane section in the simulations for endocytosis (Akamatsu et al. [2020], Serwas et al. [2022]). Thus, the cell’s morphology treated as a plane with mainly barbed end mechanics in comparison to our enclosed circular compartment of the dendritic spine head. Furthermore, prior simulation models lack filament severing based on cofilin and bending angle (McCullough et al. [2011]), which we observe can quickly increase the network size of the simulation. To accurately capture biophysical memory effects, we implement anharmonic potentials (Bader et al. [1996]) that can have multiple, spatially dependent potential wells and are designed, through DGG rules, to follow biophysical constraints and laws. Implementation within the well-founded DGG framework may also in future allow theoretical advances (Mjolsness [2013, 2019]) such as model reduction to be applied to the dendritic spine head rule system.

In this paper, we show that we can build a model for simulation of physical interaction between the actin cytoskeleton and the membrane using DGGs, and within a plausible model we can functionally characterize actin binding proteins (ABPs) that bind to and modulate individual actin monomers, affecting membrane shape. This framework can be used to grow an entire spine head from a membrane-enclosed area, though all in two dimensions for simplicity and computational tractability.

While in the past chemical kinetics have been used widely in biological simulations and DGGs have been used in simulation of microtubules (Medwedeff and Mjolsness [2023]), combining both with the biophysical kinetics of fibers and and membranes in an agent-based simulation as presented in this paper is novel. (By “biophysical kinetics”, hereinafter just “biophysics”, we refer to the dynamics of spatial positions of particles and elements of extended objects such as fibers and membranes, all due to position-dependent forces.) We consider the propulsive force of a cytoskeleton onto a membrane polygon and the respective Newtonian reaction force of the membrane onto the cytoskeleton polymers in determining the inter-module dynamics of the system. In simulating the spatial positions of the cytoskeleton, we hope to gain a better and more accurate understanding of how the actin cytoskeleton determines shape and size of a cellular protrusion.

## 2 Methods

### Methods

#### 2.1 Coarse-graining of multiple actin monomers into single objects

As actin cytoskeleton grows or shrinks within the synaptic spine head, there is a continuing turnover of F-actin governed by different dynamics at the two different ends of a single filament, the barbed and pointed ends (Pollard [1986]). The main processes that can occur in an actin network undergoing remodeling are elongation and retraction at the barbed end with rate parameters $\left(k_{\text{barbed, on}},k_{\text{barbed, off}}\right)$, elongation and retraction at the pointed end with rates $\left(k_{\text{pointed, on}},k_{\text{pointed, off}}\right)$, severing from bending angles with parameters $\left(\theta_{\text{Break, Actin}},\theta_{\text{Break, Cofilactin}},\theta_{\text{Break, Boundary}}\right)$, capping $\left(k_{\text{cap, on}},k_{\text{cap, off}}\right)$, bundling and unbundling $\left(k_{\text{CaMKII}\beta ,\text{on}},k_{\text{CaMKII}\beta ,\text{off}}\right)$, and branch nucleation $\left(k_{1},k_{\text{eq}}k_{2},k_{-1},k_{-2}\right)$ (Hotulainen and Hoogenraad [2010]). These rate parameters are all in (Table 1). We begin with rate constants found in literature and then compute the coarse-grained rate constants – replacing multiple actin monomers with 2D coordinates at their pointed end endpoints – for a more computationally efficient simulation. Using a coarse-grained model, we then simulate the dynamics of the actin cytoskeleton.

The barbed end of an actin filament adds monomers faster than the pointed end, which nets removal of monomers faster. Furthermore, the filament near the actin pointed end eventually forms more actin-ADP than near the barbed end, which retains newly added actin-ATP. These dynamics lead to directional pushing onto the membrane predominantly near clusters of barbed ends. To support this mechanism, the membrane-attached ends adaptively step, based on Brownian ratchet biophysics (Peskin et al. [1993]), their optimal coarse-graining length.

Coarse-graining is achieved by simulating aggregate rates and storing the binding numbers of attached molecules and the IDs of linked objects, for a total of 13 parameters per actin object. Below, in each DGG rule, just a relevant subset of parameters are displayed inside the 〈〈…〉〉 parentheses associated with objects.

Coarse-grained rate constants for polymerization are found by dividing the coarse-grained number into rates of elongations. The retraction, $k_{\text{barbed}}^{-}$, at a coarse-graining number is the rate for which every actin monomer, considering attached molecules, unbinds from the actin filament. Coarse-grained constants for retraction $k_{\text{barbed}}^{-}$ that are dependent on the types of actin stored inside the object are computed as a weighted harmonic mean, which equates the inverse of a summation of depolymerization times to a single rate, of an ATP-bound rate $k_{\text{barbed}}^{-}$ and an (ADP+Pi or ADP)-bound rate $k_{\text{barbed}}^{-}$ for F-actin,

$$k_{\text{barbed}}^{-}=1/\left(\frac{N_{\text{actin-ATP}}}{k_{\text{barbed-ATP}}^{-}}+\frac{N_{\text{actin-ADP}}+N_{\text{actin-ADPPi}}}{k_{\text{barbed-ADP}}^{-}}\right)$$

which is equivalent to finding the inverse of the total time it takes to depolymerize sequentially. ADP+Pi is ADP and inorganic phosphate $\left(PO_{4}\right)$ both attached to the actin object. The weights $N_{\chi }$ are the integer numbers of ATP or (ADP+Pi or ADP) monomers represented by the object. Coarse-graining methods are approximate but can significantly address the scalability of an *in silico* model which we use here.

Biophysically, we set the coarse-grained resting length as the unit length of the system. Using the unit length in meters, we scale parameters involving distance units for numerical range in simulation. Furthermore, to define a bond strength parameter of the logitudinal potential, we equate the spring constant, which is the squared-potential curvature, with the equilibrium curvature of an anharmonic Morse potential (Eq. (22)) below yielding $\alpha_{\text{Morse}}=\sqrt{\frac{k_{\text{s}}/N_{\text{CG}}}{2D_{e}}}$. The bond spring constant parameter $\alpha_{\text{Morse}}$ is in the exponent of the Morse potential. $D_{e}$ is a separately derived parameter for well-depth or equivalently known as the bond dissocation energy. Here $k_{\text{S}}$ is the experimentally measured actin spring constant, scaled as a series of springs from the original experimental measurement, into a single spring of distance between two adjacent monomers. Coarse-graining divides $k_{\text{S}}$ by the number of coarse-grained monomers, i.e. $k_{\text{s}}/N_{\text{CG}}$, in the equation defining $\alpha_{\text{Morse}}$. The angular bending constant is the flexural rigidity divided by the equilibrium length of a rod.

#### 2.2 Actin cytoskeleton remodeling

Each of the main F-actin end types, barbed and pointed, can elongate or retract based on individual rate constants that also depend on type of bound nucleotide. ADP+Pi and ADP rate constants have been found to be experimentally similar, so for simplicity we equate them in our simulation.

To provide a better picture of how a rule functions, rules for actin polymerization and retraction are shown below. In the “with” clauses we have converted the rate constant to a function of species number and membrane area instead of concentration. R(θ) is a 2 × 2 rotation matrix with angle θ.

**Actin Barbed End Elongation:**

(1) $$\;\left({◯}_{1}\to {⬤}_{2}\right)\;\langle \langle \left(x_{1},\theta_{1},\eta_{1}\right),\left(x_{2},\theta_{2},\eta_{2}\right)\rangle \rangle \;\to \left({◯}_{1}\to {◯}_{2}\to {⬤}_{3}\right)\;\langle \langle \left(x_{1},\theta_{1},\eta_{1}\right),\left(x_{2},\theta_{2},\eta_{2}\right),\left(x_{3},\theta_{3},\eta_{3}\right)\rangle \rangle \;\;\mathrm{with}\;k_{\text{barbed, on,}\eta_{3}}N_{\text{Actin, free}}\;\;\text{where}\;\left\{\begin{array}{l} \theta_{2}\sim N\left(0,\sigma_{\theta }=\sqrt{2/L_{p}}\right);\\ x_{3}=x_{2}+R\left(\theta_{2}\right)\cdot \left(x_{2}-x_{1}\right)\\ \eta_{3}\in \{\text{ATP},\text{ADP}\} \end{array}\right.$$

**Figure 1:**
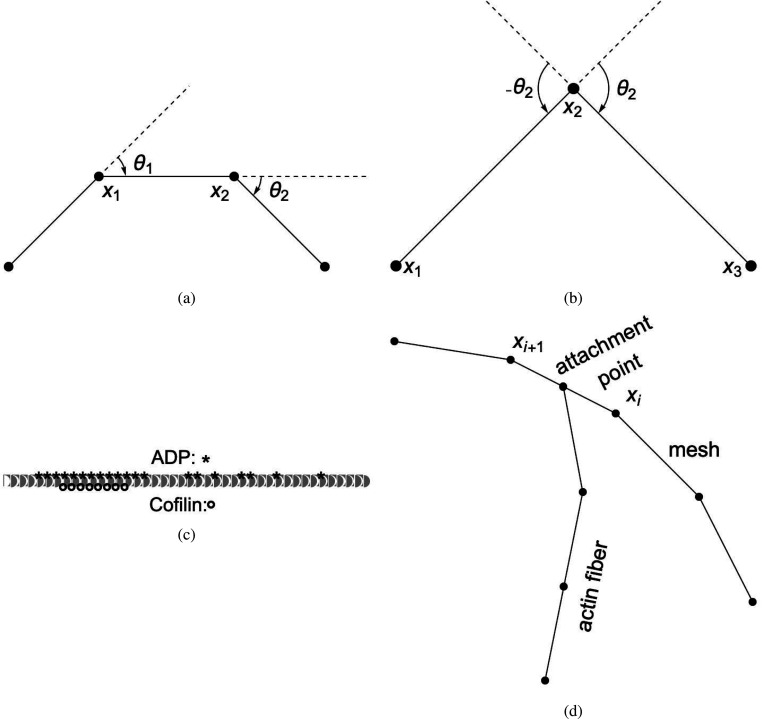
Diagrammatic visualization of the physical system with (a) illustrating the definition of angles, (b) providing opposite and equal angles with vectors, (c) visualizing an actin filament with ADP bound near cofilin boundaries, more toward the pointed end, and occasionally toward the barbed end as well as cofilin binding sequentially, and (d) depicting an actin fiber attached to a membrane mesh.

Here θ represents the middle angle between consecutive vectors of three nodes, which is zero for end nodes. The “;” represents sequential execution of two successive parameter assignments, so that $\theta_{2}$ is sampled before use in setting $x_{3}$. The sampling of $\theta_{2}$ is centered around zero as its mean, with a standard deviation $\sigma_{\theta }$ taking into account the persistence length $L_{p}$. The topology and geometric parameters of an actin fiber are illustrated in Figure 1a. As shown in the “where” clause in Eq. (1), η represents the type of nucleoside phosphate (one of ATP, ADP, or ADP+Pi) attached to the next new actin object, where we let ADP stand for both ADP and ADP+Pi due to similar rates of elongation and retraction. x, θ, and η, there are other actin object parameters to be introduced below, suppressed here for readability.

Node type symbols ◯ and ⬤ represent interior and end segments of an actin filament, each segment being one or several actin units long depending on an adjustable coarse-graining parameter. Additional node type symbols include Δ for actin network branch point junction segments, ▶ for end-capped actin segments, ■ for Arp2/3 proteins at branch points at the end of a new fiber, □ for Arp2/3 proteins at branch points not at the end of a new fiber, and symbols 🌓∈(◯,⬤,▶},⧈∈{■,□},★∈{◯,⬤,□,■,△,▶} that each represent a choice among these basic symbols. We consider graph-local rewrite rules for cytoskeleton networks, and show how to derive DGG rules from a mutually exclusive and exhaustive collection of particular graph-local neighborhood types for the left hand sides of the rules, all from a global energy function that is a sum of graph-local terms.

**Figure 2:**
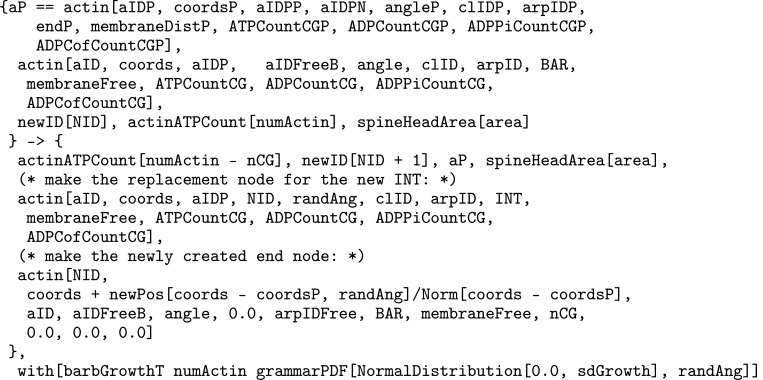
Actual Plenum rule text corresponding to the rule of Eq. (1).

The format of rule Eq. (1) above is similar to how it is implemented in Plenum with objects stored connected to one another in a graph, data stored within the objects such as position, angle, and nucleotide type, and clauses following the keyword “with” and “solving” to denote the propensity rule firing rate or differential equation respectively. The foregoing rule is presented as a mathematically idealized and “prettyprinted” version of its computational implementation in any specific piece of software. Executable rules in this paper were implemented in the Plenum package (Yosiphon [2009]) for the Mathematica computer algebra and problem-solving environment. In Plenum the foregoing rule looks like Figure 2.

From left to right, the parameters inside actin objects are an integer-valued object ID, spatial coordinates, an object ID “pointer” to the previous actin object in the filament, a pointer to the next actin object, the angle formed by the three objects, the pointer to a bundling protein, the pointer to an Arp2/3 branching object, the subtype of object e.g. end or internal, and the numbers of ATP, ADP, ADPPi, and ADPCofilin bound to the actin object. The function “newpos” rotates a vector around the origin by an angle that is sampled by another function “grammarPDF”, as in the “where” clause of Equation (1).

Similarly,

**Actin Barbed End Retraction:**

(2) $$\;\left({◯}_{1}\to {◯}_{2}\to {⬤}_{3}\right)\;\langle \langle \left(x_{1},\theta_{1},\eta_{1}\right),\left(x_{2},\theta_{2},\eta_{2}\right),\left(x_{3},\theta_{3},\eta_{3}\right)\rangle \rangle \;\to \left({◯}_{1}\to {⬤}_{2}\right)\;\langle \langle \left(x_{1},\theta_{1},\eta_{1}\right),\left(x_{2},\theta_{2},\eta_{2}\right)\rangle \rangle \;\;\mathrm{with}\;k_{\text{barbed, off,}\eta_{3}}N_{\text{Actin, free}}$$

In addition there are two further cases of the foregoing two rules with different context nodes (${□}_{1}$ and ${△}_{1}$ in addition to ${◯}_{1}$), i.e. nodes that remain unchanged in rule firing, like enzymes in a catalysed chemical reaction. Pairing the foregoing two rules together with the ⇌ bidirectional arrow, and omitting the propensity functions, these cases are:

**Actin Barbed End Elongation:**

(3) $$\;\left({□}_{1}\to {⬤}_{2}\right)\;\langle \langle \left(x_{1},\theta_{1},\eta_{1}\right),\left(x_{2},\theta_{2},\eta_{2}\right)\rangle \rangle \;⇌\left({□}_{1}\to {◯}_{2}\to {⬤}_{3}\right)\;\langle \langle \left(x_{1},\theta_{1},\eta_{1}\right),\left(x_{2},\theta_{2},\eta_{2}\right),\left(x_{3},\theta_{3},\eta_{3}\right)\rangle \rangle$$

and

**Actin Barbed End Elongation:**

(4) $$\;\left({\text{Δ}}_{1}\to {■}_{2}\right)\;\langle \langle \left(x_{1},\theta_{1},\eta_{1}\right),\left(x_{2},\theta_{2},\eta_{2}\right)\rangle \rangle \;⇌\left({\text{Δ}}_{1}\to {□}_{2}\to {⬤}_{3}\right)\;\langle \langle \left(x_{1},\theta_{1},\eta_{1}\right),\left(x_{2},\theta_{2},\eta_{2}\right),\left(x_{3},\theta_{3},\eta_{3}\right)\rangle \rangle$$

For each of these barbed end elongation/retraction rules there is a corresponding pointed end rule. The first two are shown below.

**Actin Pointed End Elongation:**

(5) $$\;\left({⬤}_{2}\to {◯}_{3}\right)\;\langle \langle \left(x_{2},\theta_{2},\eta_{2}\right),\left(x_{3},\theta_{3},\eta_{3}\right)\rangle \rangle \;⇌\left({⬤}_{1}\to {◯}_{2}\to {◯}_{3}\right)\;\langle \langle \left(x_{1},\theta_{1},\eta_{1}\right),\left(x_{2},\theta_{2},\eta_{2}\right),\left(x_{3},\theta_{3},\eta_{3}\right)\rangle \rangle \;\;\mathrm{with}\;k_{\text{pointed,}\eta_{3}}N_{\text{Actin, free}}\;\;\text{where}\;\left\{\begin{array}{l} \theta_{2}∼N\left(0,\sigma_{\theta }\right);\\ x_{1}=x_{2}+R\left(-\theta_{2}\right)\cdot \left(x_{2}-x_{3}\right)\\ \eta_{1}\in \{\text{ATP},\text{ADP}\} \end{array}\right.$$

**Actin Pointed End Retraction:**

(6) $$\;\left({⬤}_{1}\to {◯}_{2}\to {◯}_{3}\right)\;\langle \langle \left(x_{1},\theta_{1},\eta_{1}\right),\left(x_{2},\theta_{2},\eta_{2}\right),\left(x_{3},\theta_{3},\eta_{3}\right)\rangle \rangle \;⇌\left({⬤}_{2}\to {◯}_{3}\right)\;\langle \langle \left(x_{2},\theta_{2},\eta_{2}\right),\left(x_{3},\theta_{3},\eta_{3}\right)\rangle \rangle \;\;\mathrm{with}\;k_{\text{pointed,}\eta_{1}}N_{\text{Actin, free}}$$

We again have that $\sigma_{\theta }=\sqrt{\frac{2}{L_{p}}}$, where $L_{p}$ is persistence length transformed to unit length of an actin filament segment, according to the statistics of a semiflexible polymer and a random walk in small angles. Note that angle $\theta_{2}$ is being used in the reverse direction of traversal along the fiber, and hence has its sign reversed, as shown in figure 1b.

**Figure 3:**
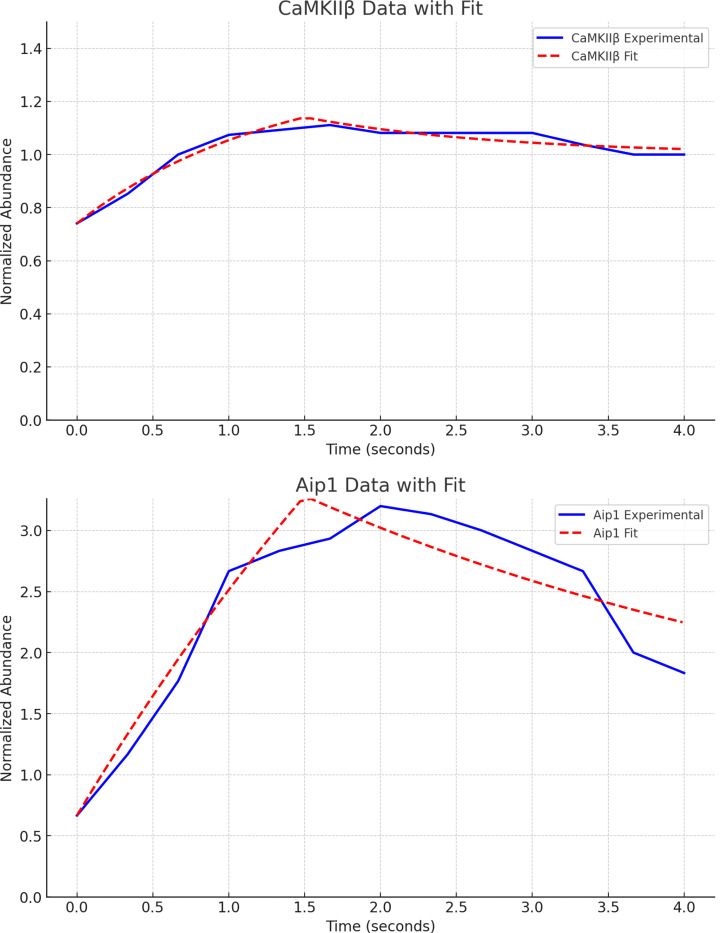
In the top plot, the estimation of CaMKIIβ synthesis and degradation rates from experimental data (Bosch et al. [2014]) is shown, and in the bottom plot, those for Aip1, a capping protein, is shown. Standard errors in estimated rates are shown with ± in Table 1 in the entries of $k_{\text{synth, cam}}$, $k_{\text{deg, cam}}$, $k_{\text{synth, cap}}$, $k_{\text{deg, cap}}$.

In addition to end elongation and retraction, there exist several more rule types for network remodeling such as:

1. Ordinary differential equation (ODE) molecule rules for synthesis and degradation and stochastic rules for phosphate release.
  Synthesis rates for CaMKIIβ and capping protein are estimated from experimental data as done previously (Bonilla-Quintana and Rangamani [2024]). As in (Bonilla-Quintana and Rangamani [2024]), the following differential equations estimate the experimental system which provides the data:
(7) $$\frac{dM}{dt}=I_{S,M}+I_{M}-k_{M}M,$$
  where M is the molecule’s normalized fluorescence measurement, I represents the influxes from stimuli $\left(I_{S,M}\right)$ and basal rates $\left(I_{M}\right)$ respectively, and $k_{M}$ is the degradation. For each molecule of interest, we fit the curves in (Bosch et al. [2014]) to differential equation Eq. (7) above. The results for CaMKIIβ (bundling) and Aip1 (capping) are shown in Figure 3 comparing the experimental data trend with the parameter fitting. The resulting estimated rates are provided in Table 1.
  The stochastic rules for phosphate release follow actin-attached nucleotide state transitions that regulate binding and unbinding of cofilin in addition to actin polymerizing dynamics. Thus, these stochastic rules regulate cytoskeletal remodeling propensities like synthesis and degradation ODE rules. Phosphate release occurs sequentially from ATP to ADP+Pi and from ADP+Pi to ADP. ADP+Pi is an intermediate state, where the inorganic phosphate (Pi) is mainly bound to the actin filament and not to the nucleotide molecule (Kudryashov and Reisler [2013]). Adjacent cofilin molecules bound to the actin filament accelerate the release of Pi from actin-ADP+Pi to yield actin-ADP.
2. Branching by Arp2/3 nucleation which forms a junction with three objects connected to a single object node with an expected angle of around $70{}^{\circ}=\frac{70\pi }{180}$ radians.
  **Actin Arp2/3 Branching:**

(8)

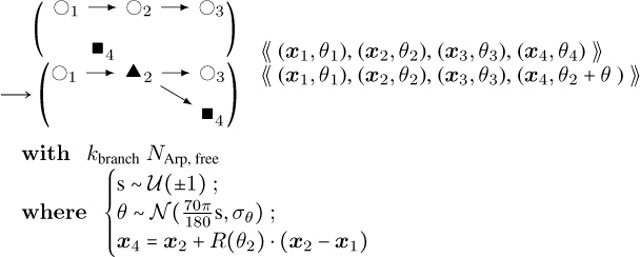

  **Actin Arp2/3 Unbranching**

(9)

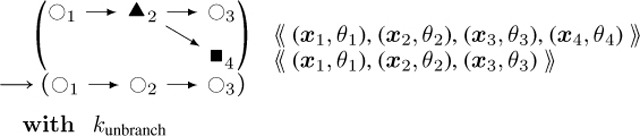

3. Capping, which stops the barbed end from elongating or retracting until the cap is removed, and uncapping:
  **Actin Barbed End Capping:**
(10) $$\;\left({◯}_{1}\to {◯}_{2}\to {⬤}_{3}\right)\;\langle \langle \left(x_{1},\theta_{1}\right),\left(x_{2},\theta_{2}\right),\left(x_{3},\theta_{3}\right)\rangle \rangle \;\to \left({◯}_{1}\to {◯}_{2}\to {▶}_{3}\right)\;\langle \langle \left(x_{1},\theta_{1}\right),\left(x_{2},\theta_{2}\right),\left(x_{3},\theta_{3}\right)\rangle \rangle \;\;\mathrm{with}\;k_{\text{cap, on}}N_{\text{Cap, Free}}$$
  **Actin Barbed End Uncapping:**
(11) $$\begin{array}{r} \left({◯}_{1}\to {◯}_{2}\to {▶}_{3}\right)\\ \to \left({◯}_{1}\to {◯}_{2}\to {⬤}_{3}\right) \end{array}\;\begin{array}{c} \langle \langle \left(x_{1},\theta_{1}\right),\left(x_{2},\theta_{2}\right),\left(x_{3},\theta_{3}\right)\rangle \rangle \\ \langle \langle \left(x_{1},\theta_{1}\right),\left(x_{2},\theta_{2}\right),\left(x_{3},\theta_{3}\right)\rangle \rangle \end{array}\;\;\mathrm{with}\;k_{\text{cap, off}}$$
4. Cofilin binding, which weakens bending stiffness of bound segments called cofilactin, and lowers the minimal angle for severing at boundaries along the actin filament (under the rule of Eq. (19) below). These rules require that further actin parameters $N_{*}$ be deployed:
  **Accelerated Cofilin Binding (1-Node):**
(12) $$\;\;({★}_{1})\;\langle \langle (x_{1},N_{\text{ADP},1},N_{\text{Cofilin},1})\rangle \rangle \;\to ({★}_{1})\;\langle \langle (x_{1},N_{\text{ADP},1}-1,N_{\text{Cofilin},1}+1)\rangle \rangle \;\;\text{with}\;k_{\text{on-edge, Cofilin}}\text{𝓗}(N_{\text{Cofilin},1}-1)N_{\text{ADP},1}N_{\text{Cofilin, Free}}$$
  **Accelerated Cofilin Binding (2-Node):**
(13) $$\;\;(\{{★}_{1}\to {\left\{★\backslash \text{⧈}\right\}}_{2})\;\langle \langle (x_{1},N_{\text{ADP},1},N_{\text{CG}}),(x_{2},N_{\text{ADP},2},0)\rangle \rangle \;\to (\{{★}_{1}\to {\left\{★\backslash \text{⧈}\right\}}_{2})\;\langle \langle (x_{1},N_{\text{ADP},1},N_{\text{CG}}),(x_{2},N_{\text{ADP},2}-1,1)\rangle \rangle \;\;with\;k_{\text{on-edge, Cofilin}}N_{\text{ADP},2}N_{\text{Cofilin, Free}}$$
  Here $N_{\text{ADP},i}$ is the number of actin proteins within filament segment object i that have attached molecules in an ADP state and $N_{\mathrm{Cofilin},i}$ is the number of actin proteins in i with attached molecules in a cofilin bound state, as illustrated in Figure 1c.
  This rule exists with its flipped version with cofilin binding from the right node to the left node towards the pointed end.
  **Bare Filament Cofilin Binding:**
(14) $$({★}_{1})\langle \langle \left(x_{1},N_{\text{ADP,1}},0\right)\rangle \rangle \to ({★}_{1})\langle \langle \left(x_{1},N_{\text{ADP,1}}-1,1\right)\rangle \rangle \;with\;k_{\text{on-single, Cofilin}}N_{\text{ADP,1}}$$
  **Actin Filament Cofilin Unbinding:**
(15) $$({★}_{1})\langle \langle \left(x_{1},N_{\text{ADP,1}},N_{\text{Cofilin,1}}\right)\rangle \rangle \to ({★}_{1})\langle \langle \left(x_{1},N_{\text{ADP,1}}+1,N_{\text{Cofilin,1}}-1\right)\rangle \rangle \;with\;k_{\text{off-edge, Cofilin}}N_{\text{Cofilin,1}}$$
5. Bundling, which creates another junction type with a CaMKIIβ object connected to two actin objects that are not already connected to each other:
  **Actin Filament CaMKIIβ Binding:**

(16)

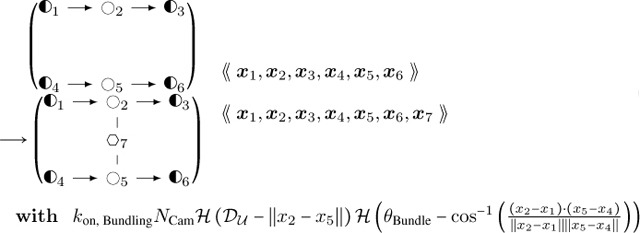


where $D_{U}$ and $\theta_{\text{Bundle}}$ are maximal numerical values of distance and angle for coarse-grained binding of CaMKIIβ, and where we denote the Heaviside function as
$$H(x)=\{\begin{array}{lll} \; & 1 & x\geq 0\\ \; & 0 & x<0 \end{array}$$
  The reverse process is given by this rule:
  **Actin Filament** CaMKIIβ **Unbinding:**

(17)

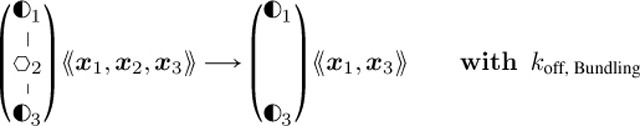

6. Bond breaking, from a large angle or large edge distance.
  **Actin Junction Breaking:**

(18)

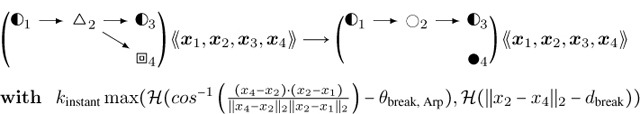


where $\theta_{\text{break, Arp}}$ and $d_{\text{break}}$ are minimal junction breaking angle difference and rod breaking distance.
  **Actin Filament Severing:**
(19) $$\;\left({◯}_{1}\to {◯}_{2}\right)\;\langle \langle \left(x_{1},\theta_{1},\eta_{\text{1, Cofilin}}\right),\left(x_{2},\theta_{2},\eta_{\text{2, Cofilin}}\right)\rangle \rangle \;\to \left({⬤}_{1}\;{⬤}_{2}\right)\;\langle \langle \left(x_{1},\theta_{1},\eta_{\text{1, Cofilin}}\right),\left(x_{2},\theta_{2},\eta_{\text{2, Cofilin}}\right)\rangle \rangle \;\;with\;k_{\text{instant}}\max(H\left(\theta_{1}-\theta_{\text{break, Actin}}\right)\delta \left(\eta_{\text{1, Cofilin}},0\right),\;\;H\left(\theta_{2}-\theta_{\text{break, Actin}}\right)\delta \left(\eta_{\text{2, Cofilin}},0\right),\;\;H\left(\theta_{1}-\theta_{\text{break, Cofilactin}}\right)H\left(\eta_{\text{1, Cofilin}}-N_{CG}/2\right),\;\;H\left(\theta_{2}-\theta_{\text{break, Cofilactin}}\right)H\left(\eta_{\text{2, Cofilin}}-N_{CG}/2\right),\;\;H\left(\theta_{1}-\theta_{\text{break, Boundary}}\right)\left(1-\delta \left(\eta_{\text{1, Cofilin}},\eta_{\text{2, Cofilin}}\right)\right),\;\;H\left(\theta_{2}-\theta_{\text{break, Boundary}}\right)\left(1-\delta \left(\eta_{\text{1, Cofilin}},\eta_{\text{2, Cofilin}}\right)\right),\;\;H\left(\left\|x_{2}-x_{1}-d_{\text{break}}\right\|\right))$$
  This maximum (equivalent to a logical disjunction i.e. “or” operation) of Heaviside functions conditions actin filament severing on three critical breaking angles dependent on the state of molecules bound to the coarse-grained object’s actin monomers. The actin breaking angle is used for zero cofilin states within bound molecules, the cofilactin breaking angle is used for internal actins a majority of which have cofilin bound molecules, and the boundary breaking angle is used elsewhere where adjacent actin objects have differing numbers of cofilin bound molecules, hence indicating a cofilactin-actin boundary.

Together, these rules lead to a treadmilling effect with turnover of actin monomers when the actin network is sufficiently large. The network preferentially moves outward in the direction of the barbed ends since the barbed end elongation rates are faster than the pointed end elongation rates. while the nodes toward the pointed ends are eventually depolymerized. In this way it was originally proposed by theorists such as Mogilner and Oster that actin can provide a force to cellular compartments by interacting with their membranes (Mogilner and Oster [2003]). Considering Newtonian reactive forces of membrane on fiber and fiber on fiber, actin can also generate other forces within the compartment, leading to internal reorganization and deformation. The mechanism with which the actin network achieves moving the membrane along with its own fibers is the subject of the next section.

### 2.3 Biomolecular mechanisms of actin-binding proteins

Based on the foregoing rules, we summarize in this section the workings of each ABP in the context of actin filament network remodeling and biophysics.

#### 2.3.1 Actin

The polymerization and depolymerization of actin are the driving mechanisms for actin dynamics in this model. In addition, a filament sub-graph that includes long rods causes an electrostatic disconnection between two adjacent actin objects as the objects exert only small radial forces on each other. The adjacency of the actin objects has its connection broken to create two new filaments (Eq. 19).

#### 2.3.2 Arp2/3

The Arp2/3 mechanism follows the mechanism posited by (Smith et al. [2013]) where Arp2/3 exists as either activated or inactivated form. In an activated form, binding to one of the sub-sites of each coarse-grained object is sufficient to nucleate a new daughter filament. In an inactivated form, a coarse-grained object requires binding to all of its Arp2/3 sub-sites in order to inactivate its branching. A junction object with a high branching bending energy nucleates the Arp2/3 branch as a new filament with a pointed end.

#### 2.3.3 Cofilin

The cofilin mechanism includes a slow binding to an unoccupied cofilin site on an actin protein within a coarse-grained actin object, which is independent of the state of the site’s neighbors on an actin filament. There also is a faster binding, accelerated by the state of the site’s neighbors (Cruz [2009]). Each bound site is mechanically weakened by a factor of five, as cofilin is known to weaken the bending stiffness of actin filaments (McCullough et al. [2008]). Nearby cofilin-bound sites accelerate the actin-bound ATP/ADP dynamics of neighboring sites.

Cofilin, when present in high numbers, destabilizes the filament by lowering its stiffness, and causes severing (Eq. 19). Cofilin binding to an empty site on an actin filament is slow; however, each bound cofilin encourages (Eq. 13) binding of cofilin to adjacent locations along the filament. Cofilin also facilitates the release of ADP+Pi to ADP when it is present near to the actin monomer and facilitates severing along interfaces between cofilin-bound segments and non-bound segments (Wioland et al. [2017]). These mechanisms are implemented in our simulations with bound ATP/ADP and cofilin stored as values in actin object nodes (Eqs. 12–15).

#### 2.3.4 CaMKIIβ

Filaments that are within a specified distance and angle of each other have a non-zero rate of binding by CaMKIIβ which links them with the the actin biophysics described in section 2.5 below. This includes the anisotropic buckling force and the bending force, together with their respective thermal Hessians, although the forces are weaker as estimated in (Kim et al. [2007]). The initial separation between the filaments is kept as the optimal length until the bundling molecule unbinds.

### 2.4 Spine head morphodynamics

Dynamical Graph Grammars (DGGs) provide a well-defined way to accommodate dynamically changing system structure such as active cytoskeleton represented using dynamic graphs within nonequilibrium statistical physics as defined by the master equation. Such structure changes operate naturally at a coarser spatial scale than the force exertion of fixed topology objects on one another in biophysical kinetics. To obtain multiscale models then it is necessary to unify the two perspectives - grammar-defined discrete state changes and finer-scale biophysics - with specialized DGG kinetics rules that obey biophysical constraints such as Galilean invariance, conservation of momentum, and dissipation of conserved global energy.

In our model the spine head area grows, starting from a small area that encloses a starting network of four actin objects with an Arp2/3 junction. The mechanism of actin-membrane mutual growth is the attachment of actin objects to the membrane mesh as soon as they intersect, allowing propulsive force onto the membrane, Newtonian reactive force onto the actin network, and pressure onto the actin network. The entire model takes place in two dimensions (viewing the dendritic spine head from the top), due to our current computational limitations, although future work will aim at three dimensions. The requisite dynamics conditions for the implementation details are provided below.

First, actin rods intersecting through a pseudo-extended actin overgrowth length (Medwedeff and Mjolsness [2023], Medwedeff [2024]) with a spine head membrane rod have the end attached to the spine head membrane rod through the creation of a new spine head membrane vertex at that coordinate. Second, the membrane is allowed to fluctuate from the curvature, pressure, and tension energy terms. The Helfrich energy mean curvature energy is given by:

$$E_{H}=\frac{\kappa }{2}\int_{S}{\left(2H\right)}^{2}dA$$

To discretize this term for a 2D membrane polygon, we approximate the mean curvature H at each vertex ${\text{r}}_{i}$. Let ${\text{r}}_{i-2},{\text{r}}_{i-1},{\text{r}}_{i+1},{\text{r}}_{i+2}$ be the neighboring vertices of ${\text{r}}_{i}$. Using the adjacent neighbors, we take the derivative of the unit tangent vector with respect to the arc length $\bar{w}$, which yields

(20) $$H_{\text{Γ}\left(x_{a},x_{b},x_{c}\right)}=\left\|\frac{d\text{T}}{d\bar{w}}\right\|,$$

where we approximate

(21) $$\frac{dT^{i}}{d{\bar{w}}^{i}}≃\frac{1}{z^{i}}\left(\frac{x_{1}^{i+1}-x_{1}^{i}}{v^{i+1}}-\frac{x_{1}^{i}-x_{1}^{i-1}}{v^{i}},\frac{x_{2}^{i+1}-x_{2}^{i}}{v^{i+1}}-\frac{x_{2}^{i}-x_{2}^{i-1}}{v^{i}}\right),$$

and $v^{i}=\left\|x^{i}-x^{i-1}\right\|$ and $z^{i}=\frac{v^{i}+v^{i+1}}{2}$. This scheme was presented in (Bonilla-Quintana et al. [2020] (Supplementary Material)), and when followed, leads to the update as follows: where $g^{i}={\left\|\frac{x^{i+1}-x^{i}}{v^{i}}-\frac{x^{i}-x^{i-1}}{v^{i-1}}\right\|}^{2}$, we have that $H_{\text{Γ}\left(x_{a},x_{b},x_{c},x_{d},x_{e}\right)}=\frac{\sqrt{g^{i-1}}}{z^{i-1}}+\frac{\sqrt{g^{i}}}{z^{i}}+\frac{\sqrt{g^{i+1}}}{z^{i+1}}$.

The actin end is allowed to interpolate from the spine head membrane after polymerizing and extending beyond the overgrowth threshold length for the overgrowth dynamics, which follows the Brownian ratchet polymerization mechanism (Peskin et al. [1993]). An interpolating membrane mesh is maintained that imparts actin-end forces onto the vertices through attachment objects connecting actin ends to particular membrane rods as in Figure 1d.

### 2.5 Actin network biophysics

The gradient descent rules below together comprise a stochastic first order Euler update for solving the force balance system of differential equations. It is analogous to stochastic gradient descent. These rules have a parameter $k_{\text{kinetic}}$ which in the infinite limit recovers the ODE exactly. Alternatively we could have coded ODEs in the DGG, but we think that would be less efficient in the presence of substantial thermal noise.

There are two sub-sectors to our implemented biophysics sector: (1) pairwise forces due to energy terms coupling two adjacent segments or proteins of a polymer (and which subserve a fiber buckling process), and (2) three-node forces that act specifically on fiber bending angles. For each of these kinds of forces, there are several rules that update position according to the force. There is also a corresponding energy term whose negative spatial derivative is the force, and another kind of rule that implements Hessian Boltzmann sampling which represents thermal noise fluctuations.

To ensure that even dissipative biophysics is compatible with conservation of energy, the DGG kinetic rules must all be derivable from a single global energy function. This function will be a sum of two-object and three-object potential energy functions. The three-object potential energies will be a function of the bending angle between three consecutive graph-connected filament segments, hence invariant to the Euclidean group of global translations and rotations. The two-object potential energies will be a function of the distance between the centroids of two graph-connected objects, hence also Euclidean-invariant.

The longitudinal pairwise energy $U_{\text{sep}}\left(x_{1},x_{2}\right)=U_{\text{sep}}(r)$, where r is the separation distance $r={\left\|x_{1}-x_{2}\right\|}_{2}$ between two connected filaments segments (which could be a small as individual actin proteins), is standardly assumed to have a minimum-energy distance $r_{\min}$, to increase for larger distances and asymptote from below to a value of zero energy, and to increase rapidly to infinity as the segment-segment pairwise distance drops to zero - the repulsive core of the central force law. For example, this intermolecular potiential energy can be taken to be the Morse potential:

(22) $${\hat{U}}_{\text{Morse}}\left(x_{1},x_{2}\right)={\hat{U}}_{\text{Morse}}\left({\left\|x_{1}-x_{2}\right\|}_{2}\right)=D_{e}{\left(1-e^{-\alpha \left(\left\|x_{1}-x_{2}\right\|-r_{\text{min}}\right)}\right)}^{2}.$$

This potential takes its minimum value at $r=r_{\min}$. We will use this popular default choice, though there is no particular reason that actin proteins should have this potential energy law rather than one of many others that have similar properties.

This kind of potential smoothly “breaks” the connection between two segments for large r, since an energy constant with respect to position implies zero force. Thus two sufficiently distant segments could trigger a filament-breaking rule such as Eq. (19).

When such a model is applied to three successive points in a fiber, the result is a tri-nodal potential in which a buckling effect arises whereby under compression the middle filament node pushes away from the co-linear state. The relevant energy combination is:

(23) $$U_{\text{tri-nodal,sep}}\left(x_{1},x_{2},x_{3}\right)={\tilde{U}}_{\text{sep}}\left(x_{1},x_{2}\right)+{\tilde{U}}_{\text{sep}}\left(x_{2},x_{3}\right)$$

For an unbranched interior actin node in a filament at position $x_{2}$, the gradient of this energy equals the gradient of the globally summed $U_{\text{sep}}$ over all connected pairs. There is a numerical reason to use the trinodal potential rather than the pairwise potential in the main DGG rule for buckling. For a nearly straight fiber, the gradients of the two pairwise potentials in which a given interior (non-boundary) node participates nearly cancel out. This cancellation is more efficiently achieved by an exact analytic vector sum in the gradient calculation of the trinodal potential, than by the stochastic addition of displacements resulting from the stochastic firing of a pairwise DGG rule on randomly chosen nodes which will eventually converge to the same stochastic cancellation on average.

Also for numerical reasons, we introduce a force-clipping two-body potential energy function that is used to ensure that the movements of the actin objects smoothly update towards a system minimum. The force-clipping is important because we are using a potential energy function $\hat{U}(r)$ for interparticle distance $r=\left\|x_{1}-x_{2}\right\|$, such as the Morse potential ${\hat{U}}_{Morse}(r)$, that includes a strong repulsive core. The singularity of high potential energy at zero distance reflects the high repulsive force if the actin objects move close enough to each other such that their electron orbitals repel strongly, but it also introduces numerical ill-conditioning.

To control this problem, we linearize the potential at distances r≤ϵ and match potential value and slope at r=ϵ, via Taylor’s theorem at that point:

(24) $$\hat{U}\left(x_{1},x_{2}\right)=\hat{U}(r)\approx {\hat{U}}_{\epsilon }(r)\equiv \hat{U}(\epsilon )+{\hat{U}}^{\prime }(\epsilon )\times \left({\left\|x_{1}-x_{2}\right\|}_{2}-\epsilon \right),$$

The resulting potential function ${\tilde{U}}_{\epsilon }(r)$ is defined piecewise as the original unclipped potential for inter-molecular distance above a threshold ϵ; below that distance it becomes a linear function without discontinuity that meets the y-intercept:

(25) $$\begin{array}{l} {\tilde{U}}_{\epsilon }(r)=\left\{\begin{array}{ll} \hat{U}(r) & r\geq \epsilon \\ {\hat{U}}_{\epsilon }(\epsilon )=\hat{U}(\epsilon )+{\left.\frac{\partial \hat{U}(r)}{\partial r}\right|}_{r=\epsilon }(r-\epsilon ) & 0\leq r\leq \epsilon \end{array}\right. \end{array}$$

and therefore we have the equation below for the clipped gradient proportional to the full, clipped force:

(26) $$\frac{\partial \tilde{U}}{\partial r}(r|\epsilon )=\left\{\begin{array}{ll} \frac{\partial \hat{U}}{\partial r}(r) & r\geq \epsilon \\ {\left.\frac{\partial \hat{U}(r)}{\partial r}\right|}_{r=\epsilon } & 0\leq r\leq \epsilon \end{array}\right.$$

where $r={\left\|x_{1}-x_{2}\right\|}_{2}$. The force is a well-defined and continuous derivative of the potential which acts to preserve Newton’s laws of motion.

If G is the graph of connected filament segments, with number of nodes |G| and symmetric adjacency matrix $\left[G_{ij}\in 0,1\right]$, then the associated global energy is

(27) $$U_{\text{radial}}=\sum_{i=1}^{\left|G\right|}\sum_{j<i}G_{ij}U_{\text{sep}}\left(x_{i},x_{j}\right).$$

If we were to ignore branch points in the actin filament network, the sum of radial separation energies would decompose over filaments indexed by F and having size |F|:

(28) $$U_{\text{radial}}=\sum_{F}\sum_{j=1}^{|F|-1}U_{\text{sep}}\left(x_{j},x_{j+1}\right)$$

Returning to the general graph case, we calculate the gradient:

(29) $$\nabla_{x_{l}}U_{\text{radial}}=\frac{1}{2}\sum_{i=1}^{\left|G\right|}\sum_{j\neq i}G_{ij}\nabla_{x_{l}}U_{\text{sep}}\left(x_{i},x_{j}\right)\;\;=\frac{1}{2}\sum_{i=1}^{\left|G\right|}\sum_{j\neq i}G_{ij}U_{\text{sep}}^{\prime }\left(x_{i},x_{j}\right)\nabla_{x_{l}}{\left\|x_{i}-x_{j}\right\|}_{2}\;\;=\frac{1}{2}\sum_{i=1}^{\left|G\right|}\sum_{j\neq i}G_{ij}U_{\text{sep}}^{\prime }\left(x_{i},x_{j}\right){\left\|x_{i}-x_{j}\right\|}_{2}^{-1}\left(x_{i}-x_{j}\right)\cdot \nabla_{x_{l}}\left(x_{i}-x_{j}\right)\;\;=\frac{1}{2}\sum_{i=1}^{\left|G\right|}\sum_{j\neq i}G_{ij}U_{\text{sep}}^{\prime }\left(x_{i},x_{j}\right){\left\|x_{i}-x_{j}\right\|}_{2}^{-1}\left(x_{i}-x_{j}\right)\cdot I\left(\delta_{li}-\delta_{lj}\right)\;\;=\frac{1}{2}\sum_{j\neq l}G_{lj}U_{\text{sep}}^{\prime }\left(x_{l},x_{j}\right){\left\|x_{l}-x_{j}\right\|}_{2}^{-1}\left(x_{l}-x_{j}\right)-\frac{1}{2}\sum_{i\neq l}G_{il}U_{\text{sep}}^{\prime }\left(x_{i},x_{l}\right){\left\|x_{i}-x_{l}\right\|}_{2}^{-1}\left(x_{i}-x_{l}\right)$$

where $\delta_{li}=1$ if l=i and 0 otherwise is the Kronecker delta function, so by symmetry, antisymmetry, and then change of index name, followed by collecting terms:

(30) $$\nabla_{x_{l}}U_{\text{radial}}=\frac{1}{2}\sum_{j\neq l}G_{lj}U_{\text{sep}}^{\prime }\left(x_{l},x_{j}\right){\left\|x_{l}-x_{j}\right\|}_{2}^{-1}\left(x_{l}-x_{j}\right)-\frac{1}{2}\sum_{i\neq l}G_{li}U_{\text{sep}}^{\prime }\left(x_{l},x_{i}\right){\left\|x_{l}-x_{i}\right\|}_{2}^{-1}\left(x_{i}-x_{l}\right)\;\;=\frac{1}{2}\sum_{j\neq l}G_{lj}U_{\text{sep}}^{\prime }\left(x_{l},x_{j}\right){\left\|x_{l}-x_{j}\right\|}_{2}^{-1}\left(x_{l}-x_{j}\right)+\frac{1}{2}\sum_{i\neq l}G_{li}U_{\text{sep}}^{\prime }\left(x_{l},x_{i}\right){\left\|x_{l}-x_{i}\right\|}_{2}^{-1}\left(x_{l}-x_{i}\right)\;\;=\frac{1}{2}\sum_{j\neq l}G_{lj}U_{\text{sep}}^{\prime }\left(x_{l},x_{j}\right){\left\|x_{l}-x_{j}\right\|}_{2}^{-1}\left(x_{l}-x_{j}\right)+\frac{1}{2}\sum_{j\neq l}G_{lj}U_{\text{sep}}^{\prime }\left(x_{l},x_{j}\right){\left\|x_{l}-x_{j}\right\|}_{2}^{-1}\left(x_{l}-x_{j}\right)\;\;=\left[\sum_{j\neq l}G_{lj}U_{\text{sep}}^{\prime }\left(x_{l},x_{j}\right){\left\|x_{l}-x_{j}\right\|}_{2}^{-1}\right]x_{l}-\left[\sum_{j\neq l}G_{lj}U_{\text{sep}}^{\prime }\left(x_{l},x_{j}\right){\left\|x_{l}-x_{j}\right\|}_{2}^{-1}x_{j}\right].$$

Let

(31) $$\psi (r)=U_{\text{sep}}^{\prime }(r)/r.$$

Then finally

(32) $$\nabla_{x_{l}}U_{\text{radial}}=\left[\sum_{j\neq l}G_{lj}\psi \left(\left\|x_{l}-x_{j}\right\|\right)\right]x_{l}-\left[\sum_{j\neq l}G_{lj}\psi \left(\left\|x_{l}-x_{j}\right\|\right)x_{j}\right].$$

This is the equation we follow in the local DGG update rules below.

One consequence of Equation (32) is

(33) $$\frac{dp}{dt}=\sum_{l}F_{l}=-\sum_{l}\nabla_{x_{l}}U_{\text{radial}}=(1-1)\left[\sum_{l}\sum_{j\neq l}G_{lj}\psi \left(x_{l}-x_{j}\right)x_{l}\right]=0,$$

conservation of the total system momentum vector.

In particular for an unbranched filament F of nonzero length |F|, Eq. (32) specializes to

(34) $$\nabla_{x_{Fl}}U_{\text{radial}}=\left(1-\delta_{l1}\right)\left(1-\delta_{l|F|}\right)\left(\left(\psi \left({\left\|x_{l}-x_{l+1}\right\|}_{2}\right)+\psi \left({\left\|x_{l-1}-x_{l}\right\|}_{2}\right)\right)x_{l}\right.\;\;\left.-\psi \left({\left\|x_{l}-x_{l+1}\right\|}_{2}\right)x_{l+1}-\psi \left({\left\|x_{l-1}-x_{l}\right\|}_{2}\right)x_{l-1}\right)\;\;+\delta_{l1}\psi \left({\left\|x_{1}-x_{2}\right\|}_{2}\right)\left(x_{1}-x_{2}\right)+\delta_{l|F|}\psi \left({\left\|x_{\left|F\right|}-x_{|F|-1}\right\|}_{2}\right)\left(x_{\left|F\right|}-x_{|F|-1}\right).$$

The summands in the sum in Equation (34) correspond to separate grammar rule diagrams centered on updating node l which is an interior node connected to two other nodes of any type (first term, with $\left(1-\delta_{l1}\right)\left(1-\delta_{l|F|}\right)$ factor), vs. an end node at the first- or last-indexed end (second and third terms respectively, with factors of $\delta_{l1}$ and $\delta_{l|F|}$ respectively) connected to one other node of any type.

Thus for an unbranched interior filament node:

(35) $$\nabla_{x_{Fl}}U_{\text{radial}}=\left(\psi \left({\left\|x_{l}-x_{l+1}\right\|}_{2}\right)+\psi \left({\left\|x_{l-1}-x_{l}\right\|}_{2}\right)\right)x_{l}-\psi \left({\left\|x_{l}-x_{l+1}\right\|}_{2}\right)x_{l+1}-\psi \left({\left\|x_{l-1}-x_{l}\right\|}_{2}\right)x_{l-1}.$$

The desired cancellation of longitudinal gradients along a nearly straight fiber will occur if $x_{l}\approx$ the weighted average of $x_{l+1}$ and $x_{l-1}$, i.e. if the segment centroids are roughly colinear and equally spaced.

Under tension, the quantity ψ tends to be positive so any deviation from colinearity will generate a restoring force $\propto -\nabla U_{\text{radial}}$ bringing the middle node $x_{l}$ back into line transversally, as well as towards equal longitudinal spacing. Under compression, ψ tends to be negative so transverse buckling will result.

To model random thermal displacements, it will also be necessary to calculate the Hessian matrix $\nabla_{Flm}^{2}$ of second derivatives. If $G_{lm}=0$ then the Hessian matrix element is zero, so the Hessian is a weighted version of the connection graph but with self-edges added. From Eq. (32) we calculate:

(36) $$\nabla_{x_{l}x_{m}}^{2}U_{\text{radial}}=\delta_{lm}\sum_{j\neq l}G_{lj}\psi \left({\left\|x_{l}-x_{j}\right\|}_{2}\right)-\left(1-\delta_{lm}\right)G_{lm}\psi \left({\left\|x_{l}-x_{m}\right\|}_{2}\right)\;\;+\sum_{j\neq l}G_{lj}\psi^{\prime }\left({\left\|x_{l}-x_{j}\right\|}_{2}\right){\left\|x_{l}-x_{j}\right\|}_{2}^{-1}\left(x_{l}-x_{j}\right)\cdot I\left(\delta_{ml}-\delta_{mj}\right)\cdot \left(x_{l}-x_{j}\right)\;\;=\delta_{lm}\sum_{j\neq l}G_{lj}\psi \left({\left\|x_{l}-x_{j}\right\|}_{2}\right)-\left(1-\delta_{lm}\right)G_{lm}\psi \left({\left\|x_{l}-x_{m}\right\|}_{2}\right)\;\;+\sum_{j\neq l}G_{lj}\psi^{\prime }\left({\left\|x_{l}-x_{j}\right\|}_{2}\right){\left\|x_{l}-x_{j}\right\|}_{2}\left(\delta_{ml}-\delta_{mj}\right)\;\;=\delta_{lm}\sum_{j\neq l}G_{lj}\left(\psi \left({\left\|x_{l}-x_{j}\right\|}_{2}\right)+\psi^{\prime }\left({\left\|x_{l}-x_{j}\right\|}_{2}\right)\left({\left\|x_{l}-x_{j}\right\|}_{2}\right)\right)\;\;-\left(1-\delta_{lm}\right)G_{lm}\left(\psi \left({\left\|x_{l}-x_{m}\right\|}_{2}\right)+\psi^{\prime }\left({\left\|x_{l}-x_{m}\right\|}_{2}\right)\left({\left\|x_{l}-x_{m}\right\|}_{2}\right)\right)$$

and finally

(37) $$\nabla_{x_{l}x_{m}}^{2}U_{\text{radial}}=\delta_{lm}\sum_{j\neq l}G_{lj}U_{\text{sep}}^{\prime \prime }\left({\left\|x_{l}-x_{j}\right\|}_{2}\right)-\left(1-\delta_{lm}\right)G_{lm}U_{\text{sep}}^{\prime \prime }\left({\left\|x_{l}-x_{m}\right\|}_{2}\right).$$

This is a weighted graph Laplacian. If all $U^{\prime \prime }$ are positive then this matrix is diagonally dominant with real eigenvalues that are all, by Gershoren’s theorem, nonnegative.

For a model of small random displacements consistent with a Boltzmann distribution with second-order (only, leaving first order effects to the gradient rules) approximation of energies, we update $x_{l}$ with the Gaussian.


(38) $$\mathrm{Pr}\left(x_{l}^{\prime }|x_{l}\right)\propto \exp\left[-{\left(x_{l}^{\prime }-x_{l}\right)}^{T}\left(\left|\sum_{j\neq l}G_{lj}U_{\text{sep}}^{\prime \prime }\left({\left\|x_{l}-x_{j}\right\|}_{2}\right)\right|I+\epsilon_{\text{sep}}^{2}I\right)\left(x_{l}^{\prime }-x_{l}\right)\right]$$

For first-order gradient dynamics we assume that, after a very brief transient ballistic behavior, our molecular-scale objects all come to force balance $F+F_{\text{drag}}=0$ with a drag force $F_{\text{drag}}=-\zeta v$, where v is object velocity through a medium of high viscosity η, and ζ depends on object geometry, but for a cylinder of radius R Stokes’ law gives $\zeta =4\pi \eta \frac{L}{\log(L/2R)+0.886}$ for perpendicular movement to the axis or $\zeta =2\pi \eta \frac{L}{\log(L/2R)+0.114}$ for parallel movement to the axis such as at the working filament ends in our simulation (Hunt et al. [1994]). Thus

(39) $$\frac{dx_{i}}{dt}=\frac{F_{i}}{\zeta }=-\frac{1}{\zeta }\nabla_{i}x_{i}.$$

Equation (39) can be a stiff ODE system for inter-molecular potentials, particularly in the highly repulsive regime of the Morse potential. Thus, we introduced the clipped potential to formulate force balance (Equation (39)) while relieving the numerical instability.

This rule in DGG implementation form is shown below.

**Interior Case Anisotropic Buckling:**

(40)

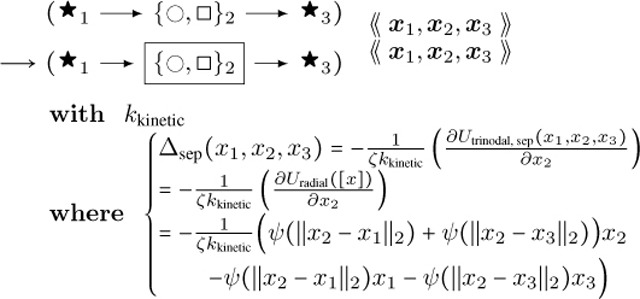




This rule effectively implements a stochastic version of a forward Euler solver for Equation (39), approaching the ODE solution as $k_{\text{kinetic}}\to \infty$. The rules of Equations 40, 59 below, and 64 below have in common that aside from the parameter values their LHS and RHS graphs are the same, and that only the parameters of the central node change, in a way that is computed systematically from the global biophysical energy function. For such rules we introduce the shorthand diagrammatic notation:

(41)

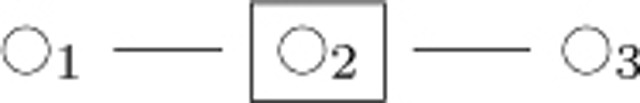


where the boxing of node 2 is not part of the graph but serves to indicate which node has changing parameter values.

Note that the empty/filled distinction is just a visualization for a binary parameter in the actual rule objects that specifies whether the given fiber segment is an end segment, i.e. has an empty binding site for continued polymerization, or not. $k_{\text{kinetic}}$ is a relative rate of updating biophysical (kinetic) rules; as it tends to infinity, there are more and more updates of smaller and smaller step sizes each per unit time, and the limit is an ordinary differential equation system.

Likewise, there is a special buckling rule for a filament branch point. It is mutually exclusive in domain of applicability with the interior case above, due to the constraints on actin segment objects in position 2. Defining similarly to Equation (23)

(42) $$U_{\text{quad-nodal, sep}}\left(x_{1},x_{2},x_{3},x_{4}\right)={\tilde{U}}_{\text{sep}}\left(x_{1},x_{2}\right)+{\tilde{U}}_{\text{sep}}\left(x_{2},x_{3}\right)++{\tilde{U}}_{\text{sep}}\left(x_{2},x_{4}\right),$$

the rule is:

**Branch Case Anisotropic Buckling:**

(43)

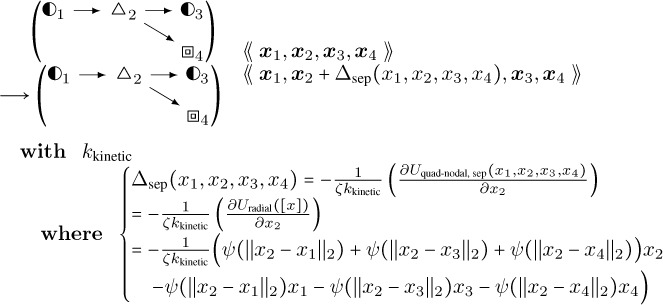




The last mutually exclusive case occurs at any filament end segment. That rule is shown in Equation (66) in Section 2.7 on filament boundary conditions.

Edges are constructed by IDs as parameters associated to objects. The forward ID is represented by the arrow, which can emanate from any object including two arrows out of a junction object to point towards an Arp2/3 object (square) as well.

Another module we add that accounts for more microscopic phenomena than the larger-scale whole ionic interactions between actin monomers is an angle bending energy that accounts for potential alignments of multiple electrostatic interaction residues between the amino acids of actin monomers. This structure imposes a constraint that the actin monomers follow the optimal bending angle created by multiple binding residues between two adjacent proteins. If the positions of three adjacent actin objects are defined as $x_{1}$, $x_{2}$, and $x_{3}$, then the angle bending potential energy is

(44) $$U_{\text{tri-nodal, ang}}\left(x_{1},x_{2},x_{3},\theta_{\text{target}}\right)=\frac{k_{\text{B}}}{2}{\left(\cos^{-1}\left(\frac{\left(x_{2}-x_{1}\right)\cdot \left(x_{3}-x_{2}\right)}{{\left\|x_{2}-x_{1}\right\|}_{2}{\left\|x_{3}-x_{2}\right\|}_{2}}\right)-\theta_{\text{target}}\right)}^{2}.$$

We define the scalar quantities

(45) $$a\left(x_{1},x_{2},x_{3}\right)=\frac{\left(x_{2}-x_{1}\right)\cdot \left(x_{3}-x_{2}\right)}{{\left\|x_{2}-x_{1}\right\|}_{2}{\left\|x_{3}-x_{2}\right\|}_{2}}\;b\left(x_{1},x_{2},x_{3}\right)=\frac{{\left\|x_{3}-x_{2}\right\|}_{2}}{{\left\|x_{2}-x_{1}\right\|}_{2}}\;c\left(x_{1},x_{2},x_{3}\right)={\left\|x_{2}-x_{1}\right\|}_{2}{\left\|x_{3}-x_{2}\right\|}_{2}$$

Note that *a* is symmetric $\left(a\left(x_{1},x_{2},x_{3}\right)=a\left(x_{1},x_{2},x_{3}\right)\right)$, as is c, but b isn’t. Parameter a=cos(θ)∈[−1,1]. For three colinear points with $x_{2}$ in the middle, the actual enclosed angle θ=0 and a=1. We define the vector quantities

(46) $$\begin{array}{l} L\left(x_{1},x_{2},x_{3}\right)=\left(x_{1}-2x_{1}+x_{3}\right)\\ \tilde{L}\left(x_{1},x_{2},x_{3}\right)=\left(bx_{1}-\left(b+\frac{1}{b}\right)x_{2}+\frac{1}{b}x_{3}\right). \end{array}$$

L is symmetric but $\tilde{L}$ is not. Then we may calculate the gradient of an interior node of three as

(47) $$\nabla_{x_{2}}U_{\text{trinodal, ang}}=-k_{\text{B}}\frac{\left(\cos^{-1}(a)-\theta_{\text{target}}\right)}{c\sqrt{1-a^{2}}}(L+a\tilde{L})$$

and the gradient of an end node of three is

(48) $$\nabla_{x_{1}}U_{\text{trinodal, ang}}=-k_{\text{B}}\frac{\left(\cos^{-1}(a)-\theta_{\text{target}}\right)}{c\sqrt{1-a^{2}}}\left(\left(x_{2}-x_{3}\right)-ab\left(x_{1}-x_{2}\right)\right).$$

Our target angles are $\theta_{\text{target}}=0$ and 70 degrees. Angles $\theta =\cos^{-1}(a)$ near 70 degrees are not a problem in the denominator but those near zero look like they might be since then a≃1. However, for $\theta_{\text{target}}=0$ and a≃1 the ratio of terms is actually nonsingular by 1’Hopital’s rule.

The corresponding interior-node Hessian as

(49) $$\nabla_{x_{2}x_{2}}^{2}U_{\text{trinodal, ang}}=H_{\text{interior}}+\left(\cos^{-1}(a)-\theta_{\text{target}}\right){\hat{H}}_{\text{interior}}\;\;H_{\text{interior}}=\frac{k_{\text{B}}}{c^{2}\left(1-a^{2}\right)}(L+a\tilde{L})⊗(L+a\tilde{L})$$

The $\left(\cos^{-1}(a)-\theta_{\text{target}}\right)\hat{H}$ term has a variable sign depending on the first factor. We will drop all such matrix terms in order to obtain a tractable, positive semidefinite approximation for use in a Gaussian distribution of position parameters for each modeled object. The resulting matrix has one nonnegative eigenvalue and (in 2D) one zero eigenvalue. We will further regularize the H matrix to make it positive definite, by taking H to be the positive eigenvalue of $H_{\text{interior}}$ times the identity matrix, or equivalently (for a rank-one matrix) the Frobenius norm or the nuclear norm of $H_{\text{interior}}$ times the identity matrix.

The full angle-bending potential energy is

(50) $$U_{\text{ang}}\left(x,\theta_{\text{target}\;ijk}\right)=\frac{k_{\text{B}}}{2}\sum_{i}\sum_{j\neq i}\sum_{k\neq j,i}G_{ij}G_{jk}U_{\text{tri-nodal, ang}}\left(x_{i},x_{j},x_{k},\theta_{\text{target}\;ijk}\right)$$

where now $G^{2}$ can be symmetrized, since any antisymmetric component will sum to zero.

We can take its gradient as

(51) $$\nabla_{l}U_{\text{ang}}\left(x,\theta_{\text{target}\;ijk}\right)=\frac{k_{\text{B}}}{2}\sum_{i}\sum_{j\neq i}\sum_{k\neq j,i}G_{ij}G_{jk}\nabla_{l}U_{\text{tri}\;ijk}\left(x_{i},x_{j},x_{k},\theta_{\text{target}\;ijk}\right)$$

(52) $$\nabla_{l}U_{\text{ang}}\left(x,\theta_{\text{target}\;ijk}\right)=-\frac{k_{\text{B}}}{2}\sum_{{\langle ijk\rangle }_{\neq }}G_{ij}G_{jk}\left(\nabla_{l}U_{\text{tri}\;ilk}\delta_{jl}+\nabla_{l}U_{\text{tri}\;ljk}\delta_{il}+\nabla_{l}U_{\text{tri}\;ijl}\delta_{kl}\right)\;\;=\frac{k_{\text{B}}}{2}\sum_{{\langle ijk\rangle }_{\neq }}G_{ij}G_{jk}\frac{\left(\cos^{-1}\left(a_{ijk}\right)-\theta_{ijk}a\right)}{c_{ijk}\sqrt{1-a_{ijk}^{2}}}[\delta_{jl}\left(L_{ijk}+a_{ijk}{\tilde{L}}_{ijk}\right)\text{​}\;\;\;+\delta_{il}\left(\left(x_{j}-x_{k}\right)-a_{ijk}b_{ijk}\left(x_{i}-x_{j}\right)\right)+\delta_{kl}\left(\left(x_{j}-x_{i}\right)-a_{kji}b_{kji}\left(x_{k}-x_{j}\right)\right)]$$

Defining

(53) $$K_{ijk}=\left(x_{j}-x_{k}\right)-a_{ijk}b_{ijk}\left(x_{i}-x_{j}\right)\;{\tilde{K}}_{ijk}=\left(x_{j}-x_{i}\right)-\frac{a_{ijk}}{b_{ijk}}\left(x_{k}-x_{j}\right)=K_{kji}$$

we have

(54) $$\begin{array}{l} \nabla_{l}U_{\text{ang}}\left(x,\theta_{\text{target}\;ijk}\right)=-\frac{k_{\text{B}}}{2}\sum_{{\langle ijk\rangle }_{\neq }}G_{ij}G_{jk}\frac{\left(\cos^{-1}\left(a_{ijk}\right)-\theta_{ijk}\right)}{c_{ijk}\sqrt{1-a_{ijk}^{2}}}\\ \;\times \left[\delta_{il}K_{ijk}+\delta_{kl}K_{kji}-\delta_{jl}\left(K_{ijk}+K_{kji}\right)\right]. \end{array}$$

$K_{ijk}$ is not symmetric under (i,j,k)↔(k,j,i), but of course $\left(K_{ijk}+K_{kji}\right)$ is. Likewise for the “usable” portion of the Hessian,

(55) $$\nabla_{lm}^{2}U_{\text{ang}}\left(x,\theta_{\text{target}\;ijk}\right)=\frac{k_{\text{B}}}{2}\sum_{{\langle ijk\rangle }_{\neq }}G_{ij}G_{jk}\frac{1}{c_{ijk}^{2}\left(1-a_{ijk}^{2}\right)}\text{​}\;\;\times \left[\delta_{il}K_{ijk}+\delta_{kl}K_{kji}-\delta_{jl}\left(K_{ijk}+K_{kji}\right)\right]\;\;\times \left[\delta_{im}K_{ijk}+\delta_{km}K_{kji}-\delta_{jm}\left(K_{ijk}+K_{kji}\right)\right]\;\;+O\left(\cos^{-1}\left(a_{ijk}\right)-\theta_{\text{target}\;ijk}\right).$$

Note that again the sum over l of this expression is zero, because the sum over l removes the Kronecker deltas from inside the first square bracket factor, leaving $K_{ijk}+K_{kji}-\left(K_{ijk}+K_{kji}\right)=0$. In the l, m space this expression is a nonnegatively weighted sum of outer products of the vectors in square brackets, so the matrix is positive semidefinite.

For efficiency in implementation we need to eliminate the Kronecker deltas. So,

(56) $$\nabla_{l}U_{\text{ang}}\left(x,\theta_{\text{target}\;ijk}\right)=-\frac{k_{\text{B}}}{2}[\sum_{{\langle jk\rangle }_{\neq ;j,k\neq l}}G_{lj}G_{jk}\frac{\left(\cos^{-1}\left(a_{ljk}\right)-\theta_{ljk}\right)}{c_{ljk}\sqrt{1-a_{ljk}^{2}}}K_{ljk}\;\;+\sum_{{\langle ij\rangle }_{\neq ;i,j\neq l}}G_{ij}G_{jl}\frac{\left(\cos^{-1}\left(a_{ijl}\right)-\theta_{ijl}\right)}{c_{ijl}\sqrt{1-a_{ijl}^{2}}}K_{lji}\;\;-\sum_{{\langle ik\rangle }_{\neq };i,k\neq l}G_{il}G_{lk}\frac{\left(\cos^{-1}\left(a_{ilk}\right)-\theta_{ilk}\right)}{c_{ilk}\sqrt{1-a_{ilk}^{2}}}\left(K_{ilk}+K_{kli}\right)].$$

Using relationships such as $\delta_{il}\delta_{im}=\delta_{lm}\delta_{il}\delta_{im}$ and, within the ${\langle ijk\rangle }_{\neq }\text{sums}$, $\delta_{il}\delta_{km}=\left(1-\delta_{lm}\right)\delta_{il}\delta_{km}$, we find a similarly reduced Hessian expression which however is the sum of nine terms rather than three. Of these nine terms, three share a factor of $\delta_{lm}$ i.e. appear on the diagonal of the Hessian, and six share a factor of $\left(1-\delta_{lm}\right)$ and are therefore off-diagonal terms. For example the diagonal terms can be calculated easily:

(57) $$\nabla_{lm}^{2}U_{\text{ang}}\left(x,\theta_{\text{target}\;ijk}\right)=\frac{k_{\text{B}}}{2}\delta_{lm}\left[\sum_{{\langle jk\rangle }_{*};j,k\neq l}G_{lj}G_{jk}\frac{1}{c_{ljk}^{2}\left(1-a_{ljk}^{2}\right)}K_{ljk}^{2}\right.\;\;+\sum_{{\langle ij\rangle }_{*};i,j\neq l}G_{ij}G_{jl}\frac{1}{c_{ijl}^{2}\left(1-a_{ijl}^{2}\right)}K_{lji}^{2}\;\;\left.+\sum_{{\langle ik\rangle }_{\neq };i,k\neq l}G_{il}G_{lk}\frac{1}{c_{ilk}^{2}\left(1-a_{ilk}^{2}\right)}{\left(K_{ilk}+K_{kli}\right)}^{2}\right]\;\;+\left(1-\delta_{lm}\right)[\ldots ]+O\left(\cos^{-1}\left(a_{ijk}\right)-\theta_{\text{target}\;ijk}\right).$$

The terms diagonal in the l, m space suffice to define a joint Gaussian model with properly nonnegative eigenvalues, but more importantly for our purpose, to define a single-l at a time Gaussian update formula similar to Equation (38).


(58) $$\mathrm{Pr}\left(x_{l}^{\prime }|x_{l}\right)\propto \exp\left[-{\left(x_{l}^{\prime }-x_{l}\right)}^{T}\right.\left.\left(\left(\sum_{{\langle jk\rangle }_{\neq }}G_{lj}G_{jk}\ldots +\ldots +\ldots \right)I+\epsilon_{\text{ang}}^{2}I\right)\left(x_{l}^{\prime }-x_{l}\right)\right]$$

The resulting DGG update rules are:

**Angle Bending 1:**

(59)

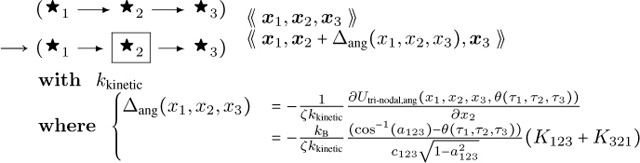




**Angle Bending 2:**

(60)

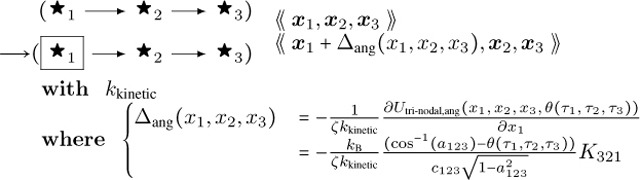




... and likewise a third rule (with the directed edges reversed) for $x_{3}$. Note that the second angle-bending rule is not exclusive to filament end cases, though the first angle-bending rule will not apply to those cases. Here $\theta \left(\tau_{1},\tau_{2},\tau_{3}\right)=\theta \left(\tau_{3},\tau_{2},\tau_{1}\right)$ is the preferred (minimal-energy) angle for three successive nodes as a function of their type data (including end-node status) τ, which we have visualized with different icons ◯, ⬤ etc..

The other module we add for biophysics is aimed at providing some thermal noise for spatial fluctuations nearequilibrium which describes continual movement of the actin filaments within the context of minimizing the Hessian of the chosen potential.

Hessian Boltzmann sampling is derived from the Taylor expansion of a potential

(61) $$U(x+u)\approx U(x)+U^{\prime }{\left(x\right)}^{T}\cdot u+{\left.\frac{1}{2}u^{T}\cdot H\right|}_{x}\cdot u+\ldots ,$$

where x is the current state, u is a displacement vector in the state, and $H=\nabla^{2}U$ is the Hessian matrix of second derivatives of U.

We extend the “heat-bath” thermal simulation algorithm to a Metropolis-Hastings version by fulfilling the following Bayesian equation for detailed balance and probability flow between two states:

(62) $$\frac{A\left(x^{\prime }|x\right)}{A\left(x|x^{\prime }\right)}=\frac{p\left(x^{\prime }\right)q\left(x|x^{\prime }\right)}{p(x)q\left(x^{\prime }|x\right)},$$

where $x^{\prime }$ is the new state and x is the old or current state, which we henceforth equate with position. q is the probability distribution for proposing a change from the old state to the new state. p is known as the target steady-state distribution to which we want the Markov Chain Monte Carlo (MCMC) algorithm to converge. For the Metropolis-Hastings algorithm the displacement proposal distributions are not homogeneous, and we analogously extend the “heat-bath” algorithm to a novel thermal noise algorithm that assigns displacement update probabilities according to the current state. The covariance of this multivariate Gaussian is the inverse of the Hessian multiplied by thermodynamic $k_{B}T$ (Das et al. [2019]). We let the mean of the sampling distribution be zero. The linear term of the Taylor expansion – assumed to be near-zero – is accounted for instead in the viscous gradient dynamics.

In this algorithm we derive a heat-bath acceptance probability for thermal noise by considering respective forward and reverse versions of the target state probabilities p(x) and proposal probabilities $q\left(x^{\prime }|x\right)$, and imposing the standard constraint of detailed balance. We consider the Boltzmann probabilities of the thermal state of a system to rely on the pure separation potential at that state. For proposal probabilities conditioned on the current state, which is our normal distribution, we take them to be proportional to the Boltzmann probability with Hessian energy term in the Taylor expansion of the current state. When the acceptance probabilities are algebraically manipulated into heat-bath form, we find that the acceptance probability takes the form

(63) $$A\left(x^{\prime }|x\right)=\frac{e^{-\frac{\Delta U-\Delta U_{q}}{k_{B}T}}\sqrt{\left|H(x)/H\left(x^{\prime }\right)\right|}}{1+e^{-\frac{\Delta U-\Delta U_{q}}{k_{B}T}}\sqrt{\left|H(x)/H\left(x^{\prime }\right)\right|}}$$

where U is the global mechanical energy, ΔU is the change in ΔU due to a local move from x to $x^{\prime }$, $U_{q}$ is just the quadratic (Hessian) part of the local Taylor expansion (Equation (61) of U, |H(x)| is the determinant of the Hessian H(x) at x, and $\Delta U_{q}\left(x^{\prime }|x\right)=U_{q}\left(x^{\prime }|x\right)-U_{q}\left(x|x^{\prime }\right)$. The constant term of course drops out under the difference Δ operation. The factors of $\sqrt{\left|H\left(x^{\prime }\right)\right|}$ arise from a Gaussian integral approximation to the partition function normalization $\int dx\exp\left(-\beta U_{q}\left(x|x^{\prime }\right)\right)$ for the heat bath move proposal $q\left(x|x^{\prime }\right)$, which depends on move starting position $x^{\prime }$.

If we instead used both the linear and quadratic terms as in Eq. 4.7 of (Lavenda [1991]), then the approximation of ΔU would be better except near the local energy minimum, leading to good acceptance ratios A. However, we opt to separate thermal noise from active drive processes in the rule set since they are physically distinct. The two options are equivalent near the local energy minimum, at thermal equilibrium or quasi-equilibrium. The probability of a “proposal” step under $\Delta U_{q}$ will be a zero-mean Gaussian or Normal distribution with precision matrix (inverse covariance matrix) $H|k_{B}T$. The acceptance of such a step will depend strongly on its alignment with the gradient where the gradient is high, but will be suitably thermal near energy minima in the manner of an Ornstein-Uhlenbeck process.

We have calculated above the relevant Hessians for both buckling and bending energies. These Hessians can be summed together or, if one is expected to be substantially smaller in a matrix norm sense than the other, only the larger one can be kept. Since the central Morse-style potentials can have high derivative values at the cores, we retain just the buckling Hessian. Then, for a change in one particular $\vec{x_{i}}$ alone,

$$\Delta_{i}U([x])=\Delta_{i}U_{\text{sep}}\left(\vec{x_{i}}|\mathrm{nbd}\left(x_{i}\right)\right),$$

i.e. only local summands of $U_{\text{sep}}$ are involved.

The DGG rule is shown below.

**Hessian Thermal Noise:**

(64)

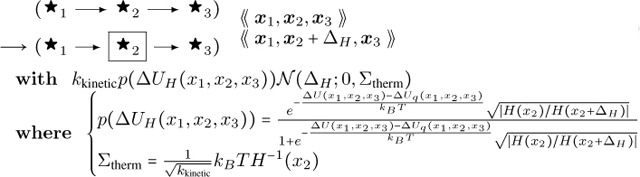




For consistency we also need to include the actin fiber end case. It is in Section 2.7 below.

Next, we explain how the dynamics of the membrane can be implemented in the DGG simulation also in the form of stochastic rules. There are three terms which comprise the total membrane energy: membrane area energy, membrane length energy, and membrane Helfrich mean curvature energy for the spine head membrane (Bonilla-Quintana et al. [2020]). The total energy is

(65) $$U_{\text{mem}}=P\Omega +\tau S+2\kappa \int_{\text{Γ}}\left(H^{2}\right)d\bar{w}.$$

Here, P is the absolute pressure difference with membrane as boundary, Ω the area, τ the line tension, κ the bending modulus, Γ the 1D representation of the manifold that is the 2D spine head membrane surface, H the mean curvature, and $\bar{w}$ the arc length (Bonilla-Quintana et al. [2021]). Mean curvature is approximated using forward finite differences.

The pressure term PΩ uses vector cross products of neighboring membrane vertex coordinates to calculate 2D area Ω, according to a discretization of the spine head polygon (Eq. 68).

Parameters used in our simulations are provided in Table 1.

### 2.6 Efficient implementation

We have approached the issue of computational efficiency in our simulation in multiple ways including parameter tuning, rule-writing, and biophysical theory.

For parameter tuning, we have chosen to set the dissociation energy as the minimum energy possible to break a single bond in an actin filament rather than to consider the distribution of energy over all bonds in the filament. This energy arises from the electrostatic dissociation energy required for one actin monomer to break away from a stable actin tetramer (Sept and McCammon [2001]). In rule-writing, we sample tri-nodal forces so that the net update is weaker in magnitude than pairwise forces, and anisotropic towards a perpendicular axis. In theory of biophysical kinetics, we derived a clipping potential Eq. (25)) that sets the force to a constant value past a multiplicative factor below the optimal length. Also, the bending energy’s gradient and Hessian are simplified to a few terms (Eqs. (56) and (57)).

### 2.7 Boundary conditions

The first boundary condition rule is for buckling. It is mutually exclusive in domain of applicability with the previous two (interior and junction node) cases.

**End Case Anistropic Buckling:**

(66)

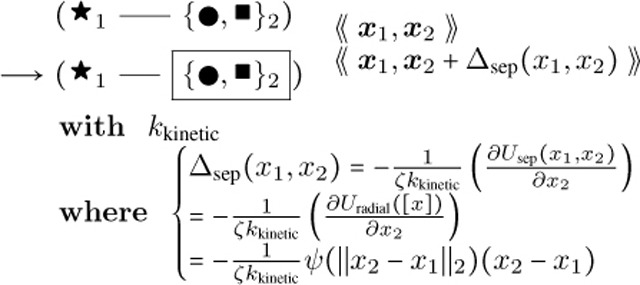



Likewise, the Hessian Thermal Noise end case is:

**End Case Hessian Thermal Noise:**

(67)

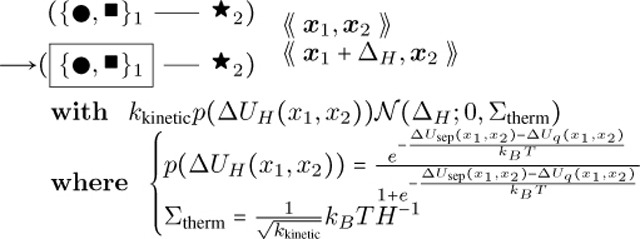




In each of these rules the undirected edge on both LHS and RHS is a shortand notation for a pair of directed-edge rules. In the first of the pair, both undirected edges are replaced with edges directed in the same left-to-right direction in the picture; and in the second of the pair, both undirected edges are replaced with edges directed in the same right-to-left direction.

## 3 Results and Discussion

We implement a DGG algorithm inside a simulator in Plenum that minimizes the biophysical energy of a dynamically changing network. The total energy function is shown below

(68) $$E_{\text{tot}}=\underset{\text{Anisotropic Buckling}}{\underset{︸}{\sum_{i,j\neq i}^{N}G_{ij}U_{\text{sep}}\left(x_{i},x_{j}\right)}}+\underset{\text{Angular Bending Energy}}{\underset{︸}{\sum_{i\neq j\neq k\neq i}^{N}G_{ij}G_{jk}U_{\text{ang}}\left(x_{i},x_{j},x_{k},\theta_{\text{target}}\right)}}\;\;\underset{\text{Membrane Areal Energy}}{\underset{︸}{+P\sum_{b,c\neq b}^{M}g_{bc}\frac{x_{b}^{x}x_{c}^{y}-x_{c}^{x}x_{b}^{y}}{2}}}+\underset{\text{Membrane Line Tension Energy}}{\underset{︸}{\Omega \sum_{b\neq c\neq d\neq b}^{M}G_{bc}G_{cd}\frac{{\left\|x_{b}-x_{c}\right\|}_{2}+{\left\|x_{c}-x_{d}\right\|}_{2}}{2}}}\;\;\underset{\text{Membrane Mean Helfrich Energy}}{\underset{︸}{+2\kappa \sum_{a\neq b\neq c\neq a}^{M}G_{ab}G_{bc}H_{\text{Γ}\left(x_{a},x_{b},x_{c}\right)}^{2}.}}$$

In this formula, the “Membrane Areal Energy” term is mediated by a directed graph with adjacency matrix $g_{bc}$ which consists of the counterclockwise cycle of edges around the 1D membrane embedded in 2D space.

**Figure 4:**
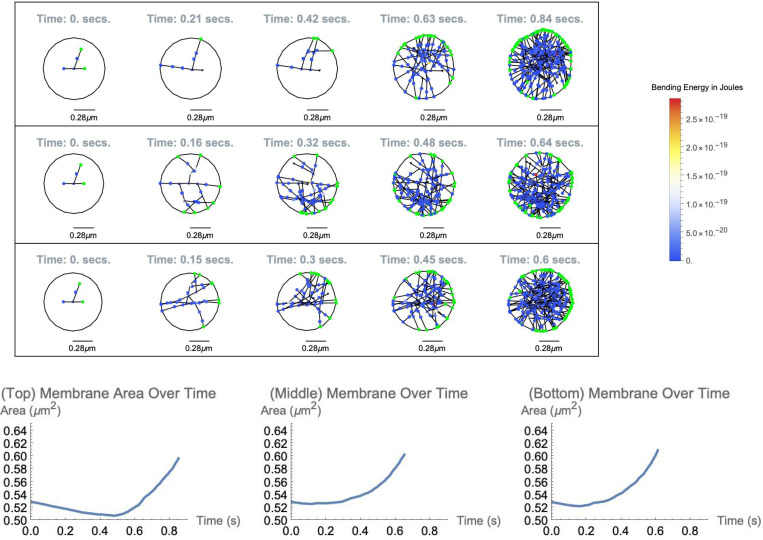
Top: Snapshots of two simulations of the synaptic spine head. Color indicates the degree of angle bending energy (or green for a barbed end) for each object, circles represent coarse-grained actin objects $\left(N_{CG}=50\right)$, triangles represent caps or colored triangles represent junction objects, and hexagons represent CaMKIIβ bundling complexes. Bottom: Membrane-enclosed area over time. Plots arranged from left-to-right correspond to simulations arranged top-to-bottom.

Simulations that run over 0.6 seconds of biological time achieve compartment growth of 20% (Fig. 4). An area of 0.5 $\mu m^{2}$ corresponds to the transverse cross-sectional area of a typical, biological synaptic spine head which indicates that a more powerful DGG simulation package that utilizes accelerated algorithm in C++ (Medwedeff and Mjolsness [2023], Medwedeff [2024]) possibly coupled with larger coarse-graining numbers would feasibly simulate changes to a synaptic spine head on a long-term scale. This would make possible the simulation of long-term potentiation (LTP) from repeated electrical signals in the form of Hebbian learning.

**Figure 5:**
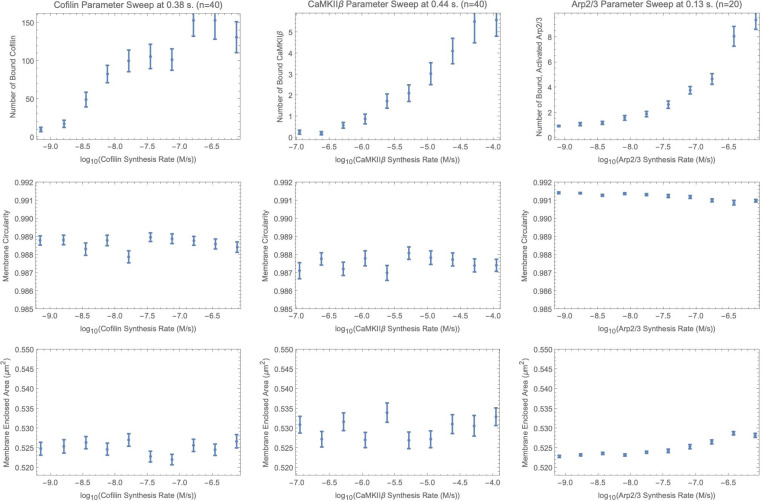
Dependence of membrane area on synthesis rates of three actin-binding proteins demonstrated in parameter sweeps. The normal synthesis rate provided in Table 1 is the middle horizontal-axis value. The three ABPs are increasingly bound to actin filaments as the synthesis rates increase (Top Row). We measure membrane circularity, defined as $\frac{4\pi \text{(membrane area)}}{{\left(\text{membrane perimeter}\right)}^{2}}$, (Middle Row) and membrane area (Bottom Row). The number of simulations is 40 for cofilin and CaMKIIβ or 20 for Arp2/3 corresponding to each data point in the plots. Error bars show standard error with Bessel’s correction. Time point is taken early for Arp2/3 because of the large, computationally demanding, network size at higher synthesis rates.

As shown in Fig. 5, we can see that cofilin concentration, as it increases, can lie in a regime of low circularity and high membrane area depending on the synthesis rate.

The trend initially starts at a high value of area (bottom-left) and decreases membrane area as cofilin concentration increases. This result is consistent with a previously explored model and experiment (Calabrese et al. [2014]). Only a small number of cofilin is needed for creating a boundary segment prone to severing. Greater numbers of cofilin weaken the bending stiffness of the filament leading to smaller membrane area.

As investigated through simulation and *in vitro* experiments in (Scheff et al. [2022]), an anisotropy can act as a biophysical memory after a number of CaMKIIβ act like steel girders resisting against compressive pressure forces. Actin filaments aligned together by chance remain as strong beams in those directions when supported by CaMKIIβ bundling, while other directions buckle more under membranous pressure. As expected by this idea, the membrane circularity decreases as more CaMKIIβ binds in Fig. 5‘s center panel. This occurs above CaMKIIβ synthesis rate of $\approx 10^{-5.5}\frac{M}{s}$, which is approximately the normal synthesis rate. The CaMKIIβ affects membrane morphology through teamwork of multiple filaments pushing to further membrane anisotropy.

The Arp2/3 synthesis rate parameter search results in a function that increases in area with the bound, activated Arp2/3 (Fig. 5). Clearly, more Arp2/3 is bound with increasing synthesis rate, and affects the membrane area past a certain number of bound Arp2/3 (top-right).

The inherent nonlinear or log-linear patterns of the system have been characterized, with several implications: (1) There may also be an advantage for model reduction of this system using artificial neural networks which have nonlinear activation functions to capture this behavior. This would result in faster predictions about synaptic spine heads in lieu of biological experiments. (2) There can be hysteresis and therefore memory as well as feedback in the biophysically mechanical nature of the system, as demonstrated in this paper. (3) The ABPs in the simulation inversely affect membrane area and circularity, showing that spine head anisotropy increases with membrane area and protein concentration in our results. Here, *in silico* complexity is an estimate of the dependencies within a biological system. Such *in silico* observations may in the future be compared to experiment, perhaps after further computational work such as the extension to three dimensions.

Molecular dynamics (MD) simulation is similar to our simulation system in that it updates positions of simulation objects, but also exhibit differences that make DGGs more suitable for spine head simulation. MD simulations are slow, simulating on at most the nanosecond or even femtosecond scale; DGGs can operate on much coarser spatial and temporal scales, and can naturally update the number and nature of chemical species or objects in the simulation. So, they work well for remodeling of the actin cytoskeleton. They also incorporate parameters associated with each object at the “agent” level which allows simulation of spatial movement and attachment of various actin-binding proteins such as Arp2/3, end-capping protein of the barbed-end, and CaMKIIβ as well as other local state variables. Furthermore, MD simulations use ballistic movement while we implement viscous dynamics to convert forces to velocity in coarse-scale models represented as DGGs, as is appropriate in a viscous medium and spatial scale on all but the fastest time scales. In this way, we aim to provide an efficient, expressive, and suitable simulation for spine head dynamics.

## 4 Conclusion

We have demonstrated how to utilize our Dynamical Graph Grammar (DGG) simulation to reveal nonlinear trends of actin cytoskeletal elements on dendritic spine head membrane expansion in 2D. The results support that there exist biophysical effects from the number of ABPs bound to actin filament networks. In particular CaMKIIβ molecules strengthen actin filaments creating strong “girders” that stabilize the network. Arp2/3’s effect on networks is enlarging the size of the synaptic spine head and decreasing the circularity. Furthermore, there can be combined effects from mechanical stiffness and network growth as shown by cofilin’s impact on membrane area.

Our graph-based simulation algorithm is able to implement this biophysical model from underlying principles. It is a highly expressive simulation system that incorporates many modules well-suited for changing both network topology and spatial or, more generally, agent-associated parameters.

There are many avenues for future work to extend our model. Additional modules can be incorporated by design of DGGs for new cytoskeletal elements. Simulations can reach the scale of the spine head receiving multiple timed signals with implementation in the Cajete and DGGML C++ simulation packages (Medwedeff and Mjolsness [2023], Medwedeff [2024], Medwedeff and Mjolsness [2024]) that may enable three-dimensional simulations, and possibly with the help of AI (Mjolsness [2019]). DGGML’s efficiency arises from symbolic methods to analyze the model and perform decomposition. The result would be exploration of long-term potentiation using graph-based actin cytoskeletal simulation in synaptic spine heads, ultimately to identify targets to treat addiction and protect and enhance memory.

## Figures and Tables

**Table 1:** Table of parameters used in DGG simulations.

Parameter	Value	Description

$k_{\text{kinetic}}$	175 $\frac{1}{\text{s}}$	Kinetics Propensity
$N_{\text{CG}}$	50 monomers	Number of Actin Monomers in a Coarse-Grained Object
T	310 K	Temperature
$k_{B}$	$1.38\times 10^{-23}\;\frac{\text{J}}{\text{K}}$	Boltzmann’s Constant
$\epsilon_{\text{Clip}}$	0.9	Clipping Factor for Morse Potential
$\theta_{\text{Arp}}$	70°	Arp2/3 Branching Angle (Mullins et al. [1998])
$k_{\text{s}}$	$0.00725\;\frac{\text{N}}{\text{m}}$	Spring Constant of Adjacent Actin Monomers (Mogilner and Oster [2003])
$k_{\text{B}}$	$5.0\times 10^{-26}\;\text{N}m^{2}$	Bending Stiffness of Actin Filament (Isambert et al. [1995])
$k_{\text{B},\text{Arp}}$	$2k_{\text{B}}\;\text{N}m^{2}$	Bending Stiffness of Arp2/3 Branch (Xu et al. [2024])
$D_{e}$	$4.81\times 10^{-20}\;\text{J}$	Dissociation Energy for Morse Potential (Hoyer et al. [2022])
α	$7.39\times 10^{-7}\;\frac{1}{\text{m}}$	Morse Spring Constant for a F-Actin Bond (Hoyer et al. [2022])
$D_{U}$	Unit Length	Binding Distance Constraint for CaMKIIβ
$\theta_{\text{Bundle}}$	15°	Bundling Angle Constraint for CaMKIIβ (Kim et al. [2007])
$d_{\text{break}}$	1.5 Unit Length	Distance Breaking Constraint for an Actin Rod
$k_{\text{B},\;\text{Cam}}$	$\frac{1}{60}\;k_{\text{B}}$	Bending Stiffness of CaMKIIβ Bundling Segment (Kim et al. [2007])
$k_{\text{s},\;\text{Cam}}$	$\frac{1}{4}\;k_{\text{s}}$	Spring Constant for Bundling Bond to F-Actin (Kim et al. [2007])
$D_{e,\;\text{Cam}}$	$9.36\times 10^{-20}\;\text{J}$	Dissociation Energy of CaMKIIβ Bundling Segment (Khan et al. [2016])
$\alpha_{\text{Cam}}$	$3.69\times 10^{-7}\;\frac{1}{\text{m}}$	Morse Spring Constant for a CaMKIIβ Bond (Khan et al. [2016])
$\sigma_{\text{A}}$	$8.0\times 10^{-9}\;\text{m}$	Diameter of Actin Molecule (Kim et al. [2007])
$\sigma_{\text{Cam}}$	$26\times 10^{-9}\;\text{m}$	Diameter of CaMKIIβ Protein (Fleming and Fleming [2018], Varadi et al. [2022])
η	$3.17\frac{\text{kg}\cdot \text{s}}{\text{m}}$	Newtonian Viscosity of Medium (Smith et al. [2007])
$r_{\text{Actin}}$	$2.76\times 10^{-9}\;\text{m}$	Rise of Actin Helix (Dominguez and Holmes [2011])
$L_{p}$	$17.7\times 10^{-6}\;\text{m}$	Persistence Length of Actin Filament (Gittes et al. [1993])
$\theta_{\text{break},\;\text{Arp}}$	25°	Breaking Angle of Arp2/3 Branch (Xu et al. [2024])
$\theta_{\text{break},\;\text{Actin}}$	57°	Breaking Angle of Bare Actin (McCullough et al. [2011])
$\theta_{\text{break, Cofilactin}}$	73°	Breaking Angle of Cofilactin (McCullough et al. [2011])
$\theta_{\text{break, Boundary}}$	31°	Breaking Angle of Actin-Cofilactin Boundary (McCullough et al. [2011])
$k_{\text{barbed, on, ATP}}$	$11.6\times 10^{6}\;\frac{1}{\text{M}\cdot \text{s}}$	Barbed End Elongation Rate Constant (ATP) (Pollard [1986])
$k_{\text{barbed, off, ATP}}$	$1.4\;\frac{1}{\text{s}}$	Barbed End Retraction Rate Constant (ATP) (Pollard [1986])
$k_{\text{pointed, on, ATP}}$	$1.3\times 10^{6}\;\frac{1}{\text{M}\cdot \text{s}}$	Pointed End Elongation Rate Constant (ATP) (Pollard [1986])
$k_{\text{pointed, off, ATP}}$	$0.81\;\frac{1}{\text{s}}$	Pointed End Retraction Rate Constant (ATP) (Pollard [1986])
$k_{\text{barbed, on, ADP}}$	$3.8\times 10^{6}\;\frac{1}{\text{M}\cdot \text{s}}$	Barbed End Elongation Rate Constant (ADP) (Pollard [1986])
$k_{\text{barbed, off, ADP}}$	$7.2\;\frac{1}{\text{s}}$	Barbed End Retraction Rate Constant (ADP) (Pollard [1986])
$k_{\text{pointed, on, ADP}}$	$0.16\times 10^{6}\;\frac{1}{\text{M}\cdot \text{s}}$	Pointed End Elongation Rate Constant (ADP) (Pollard [1986])
$k_{\text{pointed, off, ADP}}$	$0.27\;\frac{1}{\text{s}}$	Pointed End Retraction Rate Constant (ADP) (Pollard [1986])
$k_{\text{eq}}k_{2}$	$3200\;\frac{1}{\text{M}\cdot \text{s}}$	Arp2/3 Nucleation Rate (Smith et al. [2013])
$k_{1}$	$19\;\frac{1}{\text{M}\cdot \text{s}}$	Deactivated Arp2/3 Binding Rate (Smith et al. [2013])
$k_{-1}$	$0.07\;\frac{1}{\text{s}}$	Unbinding of Deactivated Arp2/3 (Smith et al. [2013])
$k_{-2}$	$0.47\;\frac{1}{\text{s}}$	Unbinding of Activated Arp2/3 (Smith et al. [2013])
$k_{\text{cap, on}}$	$2.3\times 10^{6}\;\frac{1}{\text{M}\cdot \text{s}}$	End-Capping On Rate Constant (Hayakawa et al. [2019])
$k_{\text{cap, off}}$	$9.5\times 10^{-4}\;\frac{1}{\text{s}}$	End-Capping Off Rate Constant (Hayakawa et al. [2019])
$k_{\text{CaMKII}\beta ,\;\text{on}}$	$0.5\times 10^{6}\;\frac{1}{\text{M}\cdot \text{s}}$	Binding Rate Constant of CaMKIIβ (Khan et al. [2016])
$k_{\text{CaMKII}\beta ,\text{off}}$	$0.23\;\frac{1}{\text{s}}$	Unbinding Rate Constant of CaMKIIβ (Khan et al. [2016])
$k_{\text{ATP, Hydrolysis}}$	$0.35\;\frac{\text{1}}{\text{s}}$	ATP Hydrolysis Rate Constant on Actin Filaments (Roland et al. [2008])
$k_{\text{cof, Pi}}$	$0.035\;\frac{1}{\text{s}}$	Release of Pi from Actin Filament with Nearby Cofilin (Roland et al. [2008])
$k_{\text{Pi}}$	$0.006\;\frac{1}{\text{s}}$	Release of Pi from Actin Filament (Roland et al. [2008])
$k_{\text{cof-on, edge, ADP}}$	$17\times 10^{6}\;\frac{1}{\text{M}\cdot \text{s}}$	Recruitment of Cofilin to Bound Cofilin in ADP Presence (Wioland et al. [2017])
$k_{\text{cof-on, edge, ADP+Pi}}$	$0.45\times 10^{6}\;\frac{1}{\text{M}\cdot \text{s}}$	Recruitment of Cofilin to Bound Cofilin in ADP+Pi Presence (Pandit et al. [2020])
$k_{\text{cof-off}}$	$0.7\;\frac{1}{\text{s}}$	Unbinding of Cofilin from Actin Filament (Wioland et al. [2017])
$k_{\text{single, on, Cof}}$	$10^{4}\;\frac{1}{\text{M}\cdot \text{s}}$	On Rate Constant of Isolated Cof. (Cruz [2009])
$k_{\text{synth, actin}}$	$24.4284\times 10^{-6}\;\frac{\text{M}}{\text{s}}$	Synthesis Rate of Actin (Bonilla-Quintana and Rangamani [2024])
$k_{\text{deg, actin}}$	$0.0081\;\frac{1}{\text{s}}$	Degradation Rate of Actin (Bonilla-Quintana and Rangamani [2024])
$k_{\text{synth, arp2/3}}$	$0.0255\times 10^{-6}\;\frac{\text{M}}{\text{s}}$	Synthesis Rate of Arp2/3 (Bonilla-Quintana and Rangamani [2024])
$k_{\text{deg, arp2/3}}$	$0.0013\;\frac{1}{\text{s}}$	Degradation Rate of Arp2/3 (Bonilla-Quintana and Rangamani [2024])
$k_{\text{synth, cof}}$	$0.0237\times 10^{-6}\;\frac{\text{M}}{\text{s}}$	Synthesis Rate of Cof. (Bonilla-Quintana and Rangamani [2024])
$k_{\text{deg, cof}}$	$0.0006\;\frac{\text{1}}{\text{s}}$	Synthesis Rate of Cof. (Bonilla-Quintana and Rangamani [2024])
$k_{\text{synth, cam}}$	$3.49\pm 0.53\times 10^{-6}\;\frac{\text{M}}{\text{s}}$	Synthesis Rate of CaMKIIβ (Bosch et al. [2014], Bonilla-Quintana and Rangamani [2024])
$k_{\text{deg, cam}}$	$0.013\pm 0.002\;\frac{1}{\text{s}}$	Degradation Rate of CaMKIIβ (Bosch et al. [2014], Bonilla-Quintana and Rangamani [2024])
$k_{\text{synth, cap}}$	$0.238\pm 0.054\times 10^{-6}\;\frac{\text{M}}{\text{s}}$	Synthesis Rate of End-Capping Protein (Bosch et al. [2014], Bonilla-Quintana and Rangamani [2024])
$k_{\text{deg, cam}}$	$0.004\pm 0.001\;\frac{1}{\text{s}}$	Degradation Rate of End-Capping Protein (Bosch et al. [2014], Bonilla-Quintana and Rangamani [2024])
P	$85.7143\;\text{pN}\;\mu {\text{m}}^{-2}$	Membrane Compression Strength (Bonilla-Quintana et al. [2020])
Ω	$15\;\text{pN}\;\mu {\text{m}}^{-1}$	Line Tension Strength (Bonilla-Quintana et al. [2020])
κ	$0.18\text{pN}\mu {\text{m}}^{-2}$	Membrane Curvature Strength (Bonilla-Quintana et al. [2020])
ζ	$0.002\frac{{\text{m}}^{2}}{\text{N}\cdot \text{s}}$	Membrane Viscous Update Strength (Bonilla-Quintana et al. [2020])

## Signficance

Dendritic spines are biochemical computational units found along the dendrites of neurons. In response to a stimulus from a presynaptic terminal, dendritic spine heads undergo changes to their size and shape by remodeling their actin cytoskeleton. Biophysical modeling of dendritic spine head growth can shed light on the interplay between signaling events and cytoskeletal mechanics. Here, we present a Dynamical Graph Grammar (DGG) simulation of the synaptic spine head with actin cytoskeleton determining its size. Expected trends are predicted by the model for the actin-binding proteins cofilin, CaMKIIβ, and Arp2/3 in parameter sweeps of synthesis rates. The computational efficiency of DGG due to coarse-graining and its potential for further upscaling, together with its expressive power, positions this model as a fundamental step for future work in modeling spatially complex biological systems including long-term potentiation and depression.
